# Full dynamic similarity experiments and predictive modeling of wind turbine aerodynamics across control strategies

**DOI:** 10.1093/pnasnexus/pgag303

**Published:** 2026-09-07

**Authors:** John W Kurelek, Ilan M L Upfal, Supun Pieris, Kirby S Heck, Alexander Piqué, Marcus Hultmark, Michael F Howland

**Affiliations:** Mechanical and Materials Engineering, Queen’s University, Kingston, Ontario, Canada, K7L 3N6; Civil and Environmental Engineering, Massachusetts Institute of Technology, Cambridge, MA 02139, USA; Mechanical and Materials Engineering, Queen’s University, Kingston, Ontario, Canada, K7L 3N6; Civil and Environmental Engineering, Massachusetts Institute of Technology, Cambridge, MA 02139, USA; Applied Research Laboratory, Pennsylvania State University, State College, PA 16801, USA; Mechanical and Aerospace Engineering, Princeton University, Princeton, NJ 08544, USA; Civil and Environmental Engineering, Massachusetts Institute of Technology, Cambridge, MA 02139, USA

**Keywords:** wind energy, experimental aerodynamics, flow control, predictive modeling

## Abstract

Wind turbines operate in turbulent atmospheric flow that constantly changes. In field tests, the full impacts of turbine design and operation are difficult to measure because confounding effects from atmospheric flow cannot be controlled. We address the gap in controlled experiments of wind turbines across control settings at dynamic similarity using a unique experimental framework that enables representative study of full-scale wind turbines in a laboratory-sized wind tunnel by pressurizing up to 220 atmospheres. This pressurization enables high-throughput experiments of a scaled wind turbine, at high Reynolds numbers and across yaw-misalignments and tip speed ratios relevant to commercial wind turbines. The results confirm that control strategies currently in use that were designed for turbines operating in ideal conditions without yaw misalignment should be modified, as the tip speed ratio that maximizes power depends on the yaw angle. Critically, the changes in forces and power with misalignment angle strongly depend on the tip speed ratio. We present a Unified Wind Turbine (UWT) model, which accurately predicts the behavior observed in these controlled experiments. The reliability of the UWT model to predict the synergistic effects of yaw misalignment and tip speed ratio enables its use in wind turbine design and wind farm flow control optimization to maximize the energy production and value of wind power systems. The cost-efficient experimental testbed in a pressurized wind tunnel presented herein offers a unique opportunity to study aerodynamics at dynamic similarity with large-scale, commercial turbines without many confounding effects and challenges faced in performing field tests.

Significance statement Current engineering models used for the design and control of wind power systems struggle to predict the effect of misalignment between turbines and inflow. Misalignment is ubiquitous given operation in the turbulent atmosphere and critical to wake steering flow control used to address wake effects. This knowledge gap is exacerbated by a lack of controlled experimental data at dynamic similarity that could be used to validate models. Here, we achieve dynamic similarity by using a wind tunnel pressurized up to 220 atmospheres. We confirm the dependence of wind turbine power and loads on the turbine control and we validate a fast-running, predictive aerodynamics model that can be used for wind turbine and farm design and control, with a case study estimate based on a wind farm flow model suggesting it could potentially unlock tens of thousands of dollars per turbine every year in additional revenue.

## Introduction

Renewable energy production must accelerate to meet decarbonization goals (1). In conjunction with wind power capacity growth, better design and control of wind turbines is critical to accelerate toward climate targets by enabling maximum energy generation, minimum cost, and ensured reliability (2–5). A primary bottleneck hampering improvements in the wind power models used for wind turbine and wind farm design and control is the near-complete lack of controlled experimental data at conditions representing those in the field. Field observations of wind turbine aerodynamics are riddled with uncertainties due to lack of control over inflow and operating conditions, as well as the inability to measure fluid flows and forces accurately and with high enough resolution in the field (6–13). This limits conclusions that can be drawn from field observations, and even more so limits our ability to stress-test models.

A persistent uncertainty in wind power aerodynamics modeling and corresponding energy predictions is the effect of misalignment between the rotor and inflow. Misalignment is ubiquitous for operating turbines (14, 15), has a significant impact on power and loads (9, 16, 17), and is critical to flow control methods such as wake steering (12). Wake steering, where the yaw control mechanisms available to individual wind turbines are synergistically combined using intentional misalignment to prioritize farm-level efficiency over individual output, has been shown to be theoretically viable (18, 19). Initial field experiments at commercial wind farms have demonstrated substantial opportunity to increase power production by alleviating wake interactions between turbines (8, 11, 20). Wake steering flow control is predicted to have the potential to boost wind power economics by up to US$13,000 per MW of rated capacity per year and can reduce the footprint of wind farms, further driving wind energy deployment (21). Further research has indicated substantial potential to further improve wake steering. Munters and Meyers (22, 23) used direct optimization within large eddy simulations to reveal the synergistic potential of combining thrust (induction) control and wake steering (reviewed by Houck (24)). The field experiment reported in Howland et al. (9) demonstrated that the power reduction from yaw misalignment depends on how other variables, such as tip speed ratio or pitch angles, are controlled when it is yaw misaligned. Realizing the full potential of wake steering is critically dependent on improving the accuracy and reducing the uncertainty of the aerodynamic models used for optimization (12).

Historically, the effects of misalignment are modeled using empiricism (18, 25, 26). This knowledge gap is furthered by data limitations. Yaw misalignment is challenging to measure in field tests, as wind direction sensors are placed on the nacelle and are affected by turbine operation (9, 27). Even when misalignment is reliably measured in field tests, it is difficult to clearly parse its impact on power and loads due to the lack of control over confounding variables, such as wind speed, turbine rotational speed, and wind shear (9, 16). These challenges with field tests motivate investigating yaw misalignment, and other turbine control variables such as rotational speed, in wind tunnel tests and in simulations.

Heck et al. (28) used insights from large eddy simulations to derive a theoretical model that generalizes momentum theory to represent the aerodynamics of misaligned rotors. Heck et al. (28) also demonstrated, using both the generalized momentum theory and large eddy simulations, that the local thrust coefficient (thrust coefficient based on velocity at the rotor) that maximizes the turbine power production increases with increasing yaw misalignment magnitude. For a rotating wind turbine, the thrust coefficient is controlled by the blade pitch angles and the rotor tip speed ratio, so it is hypothesized that pitch, tip speed ratio, or both should be modified in yaw (9, 28). Subsequent modeling advances then coupled momentum theory, which accounts for yaw misalignment, with variants of blade element modeling to predict the thrust coefficient, revealing the effects of the turbine blade pitch and tip speed ratio on power and loads of misaligned turbines (29, 30). Specifically, Liew et al. (29) combined the induction model of Heck et al. (28) with a blade element model and showed that the realized power-yaw relationship inherently depends on how the pitch angles and the tip speed ratio are varied in yaw misalignment. Tamaro et al. (30) combined the same yawed induction model with an approximate, fast-running blade element model and identified that the power maximizing pitch and tip speed ratio depends on yaw misalignment, consistent with the yaw-dependent optimal thrust coefficient findings from the induction modeling and large eddy simulations of Heck et al. (28). Further, Tamaro et al. (30) presented large eddy simulations and experiments in a traditional atmospheric pressure wind tunnel of a small rotational wind turbine model across yaw, pitch, and tip speed ratios to validate their model. However, the low Reynolds numbers at the blade airfoils introduce sensitivity in the aerodynamics of the turbine to the operating conditions, and Tamaro et al. (30) calibrated aerodynamic parameters used in their model to the experimental measurements. All of the models described above and their corresponding optimal control predictions remain unvalidated using experimental data at full dynamic similarity where Reynolds number independence can be achieved. This uncertainty in the reliability of the experimental similitude is because wind tunnel experiments have historically been performed at Reynolds numbers that are orders of magnitude lower than those of contemporary wind turbines.

To reproduce the physics of the real-world fluid-structure system, lab tests must be performed at dynamic similarity, which is achieved by matching the governing nondimensional parameters (31). Preserving dynamic similarity for wind turbines requires matching the field-scale Reynolds number, ReD=U∞D/ν, tip speed ratio, $\lambda =\Omega D/2U_{\infty }$, and Mach number, $\mathrm{Ma}=U_{\infty }/c_{s}$. The freestream wind speed is $U_{\infty }$, *D* is the rotor diameter, *ν* is the fluid kinematic viscosity, *Ω* is the angular velocity of the rotor, and $c_{s}$ is the speed of sound. Modern utility-scale turbines are of O(100 m) in diameter (32), operating between 107≤ReD≤108 and 4≤λ≤10 in incompressible flow (Ma≤0.3) (33, 34).

Scaled experiments in conventional wind tunnels require turbines that are <10 m in size, two orders of magnitude smaller than field-scale turbines, and therefore cannot simultaneously satisfy all three governing parameters. To match the Reynolds number in a conventional wind tunnel, the geometric scaling requires a commensurate increase in flow velocity, in turn leading to compressibility effects due to high Mach numbers. Increasing flow speed for a small-scale model also necessitates extremely high rotation rates for the lab-scale turbine to match the tip speed ratio, making these tests practically impossible due to large centrifugal forces (35). As a consequence, previous experimental efforts have largely disregarded aspects of dynamical scaling, especially with respect to Reynolds number and tip speed ratio (36–38). An exception is Snel et al. (39), who considered effects of tip speed ratio, yaw angle, *γ*, and blade pitch angle on rotor loading and wake development at ReD=3 to 9 million. However, due to the very high costs associated with using a large three-bladed, 4.5 m-diameter turbine, tests were limited to three yaw misalignment angles and could only capture general trends in performance with wind direction. Meanwhile, velocity in the wake and turbine performance have been shown to be sensitive to Reynolds number (40–43), and a minimum threshold for Reynolds number independence remains an open problem, with observations disagreeing by more than an order of magnitude (44, 45). This is especially important given the well-established Reynolds number dependence of airfoils (46, 47), which provide the lift needed for turbine power generation.

Separately, studies employing computational fluid dynamics (i.e. large eddy simulations) struggle to capture the extreme range of scales needed to simultaneously resolve flow physics of the atmospheric wind and the boundary layer on blade airfoils (2, 48), the latter requiring micrometer resolution (49). Thus, simulations resolving the geometry of a single wind turbine rotor require substantial supercomputer effort, and themselves rely on approximate turbulence modeling closures (50). Instead, most wind turbine simulations use simplified actuator disk (51, 52) or actuator line (53, 54) formulations that parametrize, rather than resolve, the rotor aerodynamics, and use many simplifying assumptions in doing so. On the other hand, field experiments using full-scale wind turbines offer the opportunity to study the real system, but numerous challenges emerge in performing controlled experiments in the chaotic, turbulent atmospheric flow. For example, the effects of wind turbine control are often challenging to isolate in field experiments given variations in atmospheric conditions (9, 13, 16, 55, 56). This obstacle has hindered model development and introduces uncertainty in model validation.

Our understanding of wind turbine aerodynamics therefore still relies largely on tests carried out at significantly lower Reynolds numbers ($10^{4}$–$10^{5}$) (30, 36, 43), in noisy field environments (9, 16), and using approximate laboratory and in silico setups (43, 54). Bridging the gap between laboratory results (which lack dynamic similarity), field operation (which lacks control), and model predictions can only be addressed by solving the scaling issue.

This study addresses the critical bottleneck in dynamically similar experiments for wind power across control settings using high-throughput, cost-efficient lab experiments in a variable density wind tunnel at pressures up to 220 atmospheres. This high-density test environment enables Reynolds numbers almost 200 times larger than standard wind tunnels. These unique experiments provide unprecedented insights into the performance of wind turbines across control strategies at near-operational Reynolds numbers, tip speed ratios, and Mach numbers. The experiments demonstrate the dependence of yawed turbine power and forces on the turbine control (tip speed ratio), resulting from nonlinear, high Reynolds number aerodynamics. Currently, wind turbines nominally operate at a constant, target tip speed ratio when the turbine seeks to maximize power capture. This study confirms at dynamic similarity that the power maximizing tip speed ratio depends on the misalignment angle, therefore offering a new opportunity to increase energy production for modern wind turbines that are always in some degree of misalignment, as well as opportunity for additional gains from wake steering. A unified wind turbine (UWT) model, which is a fast-running predictive model for the power and forces of a wind turbine rotor across operational regimes without empirical corrections, is compared to the experimental data. The UWT model accurately predicts the increasing loss in thrust and power from yaw misalignment as a function of the tip speed ratio. Critically, the UWT model also predicts the power maximizing control strategy for each yaw angle to within experimental uncertainty. Together, this joint experimental and theoretical study widely explores the parameter space of wind turbine control at dynamic similarity, enabling thorough validation of a new model that can be reliably used for design and control optimization. Wind farm flow control using the validated UWT model unlocks new opportunities to increase performance by synergistically optimizing yaw misalignment, blade pitch angles, and tip speed ratio and, based on simplified engineering wind flow model simulations herein, could result in up to 40% higher energy gain than control using a conventional cosine model for yaw.

## Wind tunnel experimental results at dynamic similarity

We investigate the power production and thrust force of a laboratory-scale wind turbine across yaw misalignments and tip speed ratios at a Reynolds number of ReD=4×106. To maintain dynamic similarity and thus achieve field-relevant flow conditions, all experiments were conducted in the high Reynolds number test facility (HRTF) at Princeton University. The HRTF, shown in Fig. 1a, is a specialized wind tunnel that uses pressurized air as the working fluid. The facility can be pressurized up to 24 MPa and generate flow velocities up to 10 ms^−1^ (57). A 15 cm-diameter, self-starting wind turbine model was mounted in the test section of the HRTF (Fig. 1b). The experimental setup is described in Methods.

**Figure 1:**
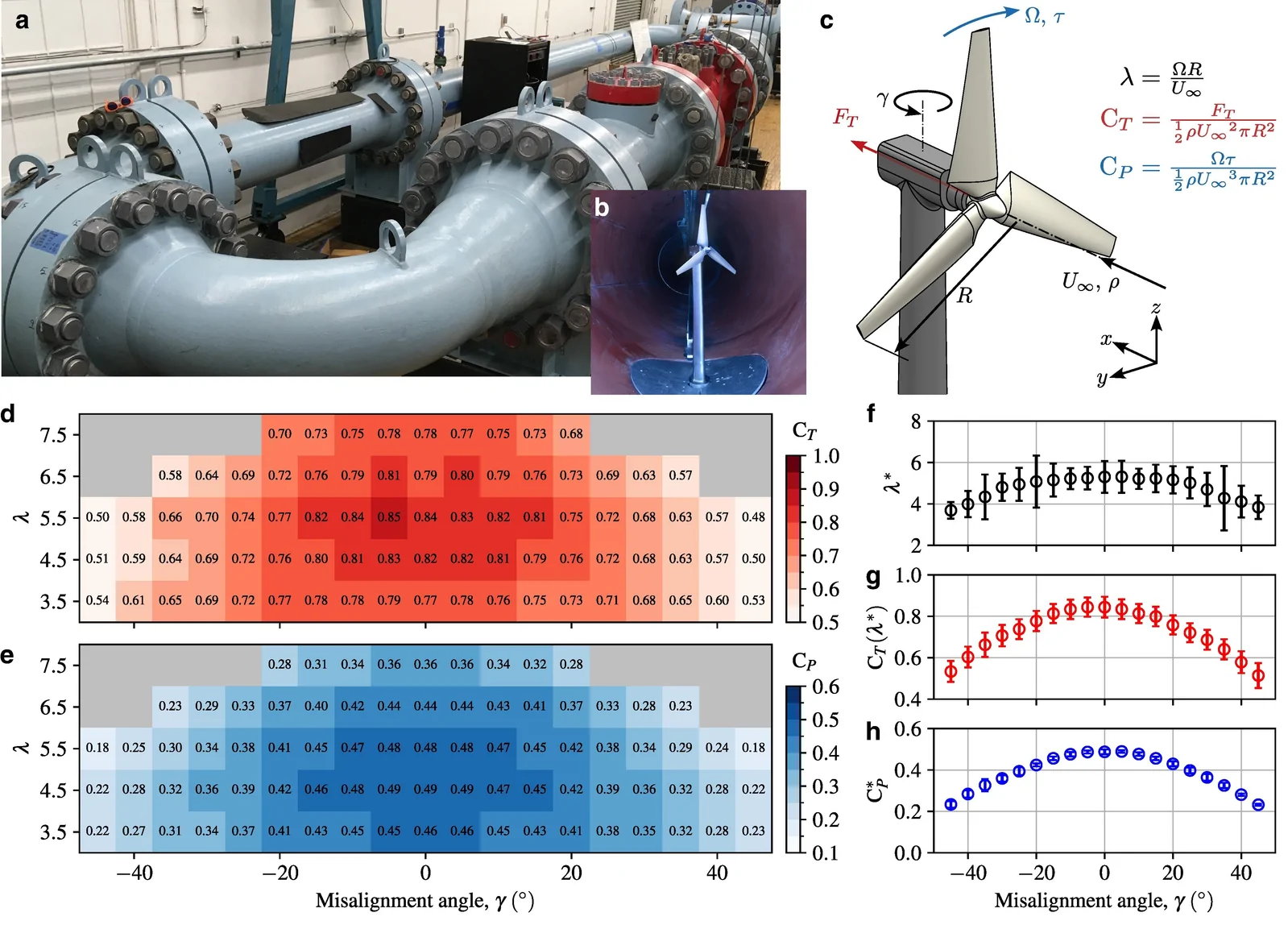
Experimental setup and results. a) Photo of the HRTF at Princeton University, which allows for density to be scaled via pressurization facilitating full-dynamic similarity to be maintained. b) Photo of the wind turbine model in the HRTF yaw-misaligned to the incoming flow. c) Schematic of the wind turbine model indicating relevant parameters and derived quantities: freestream velocity, $U_{\infty }$, density, *ρ*, rotor radius, *R*, rotor angular velocity, *Ω*, rotor torque, *τ*, misalignment angle (yaw), *γ*, rotor normal thrust force, $F_{T}$, tip speed ratio, *λ*, coefficient of thrust, $C_{T}$, and coefficient of power, $C_{P}$ along with the coordinate system. Each blade consists of a circular hub, a blended transition to NACA 63–235, then to NACA 63–214 at the tip. d) Contour plot of $C_{T}$ for all test conditions. e) Contour plot of $C_{P}$ for all test conditions. f) $\lambda^{*}$, *λ* value that maximizes $C_{P}$ for each yaw angle. g) $C_{T}$ measured at $\lambda^{*}$ for each yaw angle. h) Maximum coefficient of power at each yaw angle, $C_{P}^{*}$, measured at $\lambda^{*}$ .

The wind turbine performance is characterized by the nondimensional coefficients of thrust, $C_{T}$, and power, $C_{P}$ (Fig. 1c). The effects of wind turbine control, through tip speed ratio and yaw misalignment regulation, on $C_{T}$ and $C_{P}$ are presented in Fig. 1d and e, respectively. When holding tip speed ratio fixed, increased yaw misalignment results in decreases in $C_{T}$ and $C_{P}$ for all *λ* tested. When the turbine is yaw aligned, there is an intermediate tip speed ratio value that yields the highest power coefficient (see e.g. Burton et al. (58)). Most importantly, these results identify a distinct value of the optimal tip speed ratio which maximizes power production at each independent yaw misalignment angle. We denote this optimal tip speed ratio that depends on the yaw misalignment angle as $\lambda^{*}$.

In current wind farm flow control approaches, modification of the tip speed ratio (or pitch angles) are rarely made in conjunction with changes to yaw misalignment (12, 24). These two wind farm flow control strategies, called induction control for tip speed ratio or pitch changes and wake steering for yaw changes, are primarily considered independently. The yaw-dependent optimal tip speed ratio identified in these experiments uncovers a new opportunity for combined tip speed ratio and yaw control to yield additional value to wind farm flow control. In tandem, correctly predicting the power-yaw and thrust-yaw relationships and their dependence on tip speed ratio is critical to effective wake-steering control. Separately from flow control optimization, wind turbines almost always operate in some degree of yaw misalignment due to slow yaw controller reaction times. In contrast, the tip speed ratio (controlled by generator torque) and the blade pitch angles can be controlled much more rapidly. Therefore, the present results suggest that turbines could dynamically change their tip speed ratios to track $\lambda^{*}$ and reduce power loss when they detect operation in yaw misalignment.

We determine the optimal tip speed ratio $\lambda^{*}$ at each yaw angle based on a polynomial fit to $C_{P}$ from the initial test matrix. These optimal tip speed ratios are plotted in Fig. 1f. Another set of measurements is conducted at the optimal tip speed ratios $\lambda^{*}$ across the full range of *γ* and the corresponding optimal $C_{T}$ and $C_{P}$, denoted as $C_{T}^{*}$ and $C_{P}^{*}$, are presented in Fig. 1g and h, respectively. These low-cost, scaled, and highly-controlled experiments identify the power maximizing control strategy of a wind turbine at operational conditions (ReD, *λ*, and Ma) relevant to commercial-scale wind turbines.

## Synthesis and validation of a UWT model

The basis of fast-running aerodynamic modeling for wind turbines is classical, 1D momentum theory (59). The theory, which represents wind turbines as porous actuator disks that impose a thrust force on the incoming wind flow, yields the theoretical estimate for the maximum wind power extraction derived by Betz (60) and Lanchester (61). To yield more realistic predictions of wind turbine power and loads that account for the aerodynamics of the rotating blades and turbine control, momentum theory can be coupled to blade element modeling that computes the lift and drag on the airfoils based on the velocity incident to the airfoil which is output from momentum theory (26). This approach is called blade element momentum (BEM) modeling. However, classical 1D momentum theory cannot predict the effects of yaw misalignment, and also yields substantial errors at high thrust coefficients associated with larger tip speed ratios or lower pitch angles (62). The primary strategy to handle these two issues is to apply empirical corrections in BEM models. Recently, a Unified Momentum Model (63) was proposed to predict the power, thrust, and wakes of actuator disks for arbitrary operational strategies. This study couples the Unified Momentum Model (63) with a blade element model that represents the aerodynamic characteristics of the experimental wind turbine to yield a UWT model that does not rely on empirical yaw or high thrust corrections. The UWT model is described in Methods. A schematic of the model is shown in Fig. 2a. The UWT model is a blade element momentum approach that uses the Unified Momentum Model (63), extended to account for confinement (64), as its induction model along with a standard blade element modeling closure (26). Given the use of the Unified Momentum Model (63), the UWT can make predictions across yaw misalignments and arbitrary thrust coefficients without relying on empirical induction corrections commonly used in BEM modeling, such as skewed wake and high thrust corrections (26). The Unified Momentum Model (63) was generalized from the yawed induction model of Heck et al. (28) to account for high thrust dynamics. This approach is distinct from that of Tamaro et al. (30), whose fast-running model for yawed wind turbines uses the same induction closure of Heck et al. (28), but, in contrast, is coupled with an engineering approximation to blade element modeling to predict thrust. The UWT is compared to the model of Tamaro et al. (30) in the SI Appendix, showing that the Tamaro et al. (30) model incurs consistently higher error than the UWT model when compared to the present HRTF data, but is more computationally efficient than the UWT due to its engineering approximations of the induction and blade element models.

**Figure 2:**
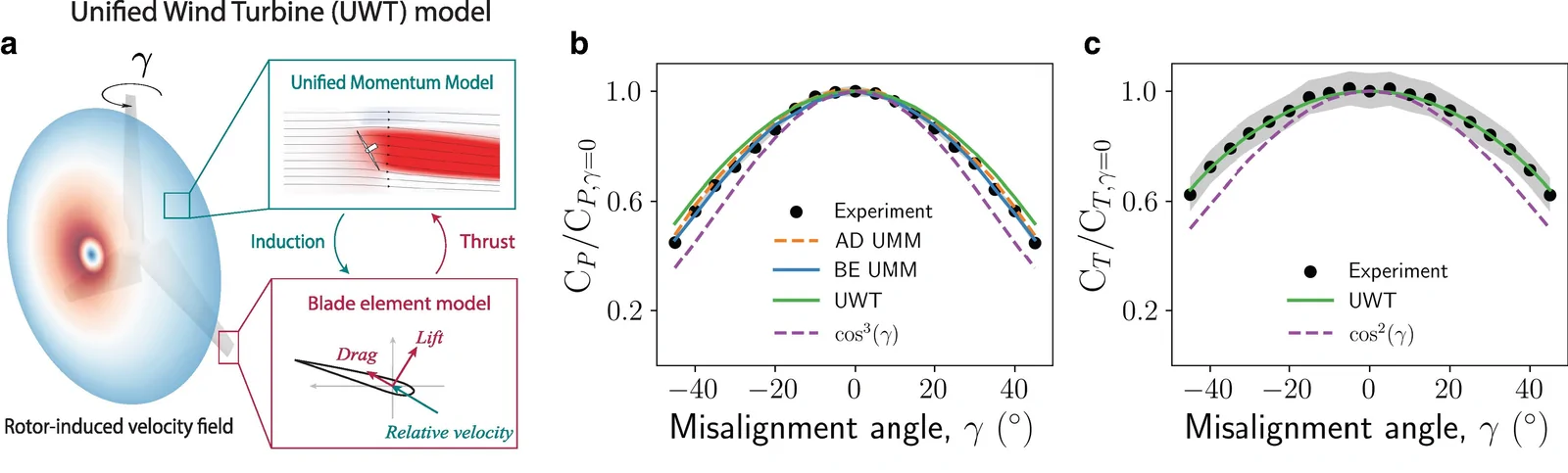
The UWT model is compared to controlled experiments at dynamic similarity to commercial wind turbines. a) The UWT model is developed by coupling the Unified Momentum Model with a blade element model. b) Power coefficient normalized by the yaw aligned value for a tip speed ratio of 4.5, comparing the UWT model to the experiments. The green line (UWT) shows the fully predictive UWT result without knowledge of the measured $C_{T}$. The blue line (BE UMM) shows $C_{P}$ computed by providing the measured $C_{T}$, revealing the unified model performance without the error incurred by the blade element model coupling. The dashed orange line (AD UMM) is the actuator disk power ($P={\vec{F}}_{T}\cdot {\vec{u}}_{d}$) from the Unified Momentum Model using the measured $C_{T}$. c) Same as (b) but for thrust coefficient.

First, we compare the UWT model to the experimentally measured thrust and power coefficients for a tip speed ratio of 4.5 across yaw misalignment angles. The high-Reynolds number nature of the experimental data used here is essential for reliable validation of the UWT because it removes a major confounding factor associated with the Reynolds number sensitivity of airfoil lift and drag coefficients that are input to wind power aerodynamics models such as BEM. In contrast, low Reynolds number experimental data would present an additional confounding factor through increased uncertainty in the aerodynamic lift and drag inputs for the UWT model. The standard approach to predict thrust and power of a yawed wind turbine is empirical cosine modeling (18, 20), $C_{T}/C_{T}(\gamma =0)=\cos^{T_{p}}\;(\gamma )$ and $C_{P}/C_{P}(\gamma =0)=\cos^{P_{p}}\;(\gamma )$. Using geometry for the rotor-normal component of the inflow velocity yields $C_{T}/C_{T}(\gamma =0)=\cos^{2}\;(\gamma )$ and $C_{P}/C_{P}(\gamma =0)=\cos^{3}\;(\gamma )$ (15, 58) and therefore these simple models are shown as a reference. Note that more precise values of $T_{p}$ and $P_{p}$ require empirical data that quantifies the effect of yaw, which has limited value for out-of-sample predictions (9). Instead, the UWT model predicts $C_{T}$ and $C_{P}$ without empiricism, yielding low errors for power compared to the experimental measurements. The slight over-prediction of the normalized coefficient of power at high yaw angles is the result of the blade element model over-predicting the magnitude of the thrust coefficient, which in turn predicts less power reduction as a function of yaw misalignment (28); this is further detailed in the SI Appendix. As reviewed by Sørensen (65), primary sources of uncertainty in BEM modeling stem from the input airfoil lift and drag coefficients and from tip-loss modeling. Future work should perform detailed characterization of the real tip losses of the rotor used in these experiments. Such a characterization would help inform better modeling choices and reduce uncertainty in predictions of the magnitude of the thrust coefficient. We also show predictions from the Unified Momentum Model when it is provided the experimentally measured thrust, thereby removing error stemming from the blade element model and yielding errors that are less than the experimental uncertainty for all yaw angles (Fig. 2b). This evaluation of the Unified Momentum Model in isolation requires input thrust coefficients, and is therefore not a full prediction, but it does indicate that the primary cause of model error in the fully predictive UWT stems from the blade element closure for the magnitude of the thrust coefficient. The normalized thrust coefficient predictions from the fully predictive UWT in Fig. 2c are also within the experimental uncertainty for all yaw angles.

Critically, the present experiments at dynamic similarity confirm that the power maximizing tip speed ratio, and correspondingly the optimal power production, depends on the yaw misalignment angle (28–30). The existing practice of empirical cosine modeling cannot predict this maximum power, nor the value of the tip speed ratio that maximizes power. In Fig. 3a and b, we demonstrate that the UWT model reliably predicts the power maximizing control strategy at each yaw angle ($\lambda^{*}$) to within experimental uncertainty and also predicts the optimal power ($C_{P}^{*}/C_{P}^{*}(\gamma =0)$) to within experimental uncertainty for $-30^{\circ }\leq \gamma \leq 30^{\circ }$, with $\gamma =-20^{\circ }$ as an outlier exception. There is some apparent difference in the curvature of the $\lambda^{*}$ results between the experiments and the UWT, which could be associated with the overestimation of the magnitude of the thrust coefficient by the blade element model used here. This predictive capability offers a new opportunity to design controllers for individual turbines and also for wind farm flow control that result in optimal operation when the turbine is misaligned with the inflow without needing costly field experiments to determine the optimal control setpoints.

**Figure 3:**
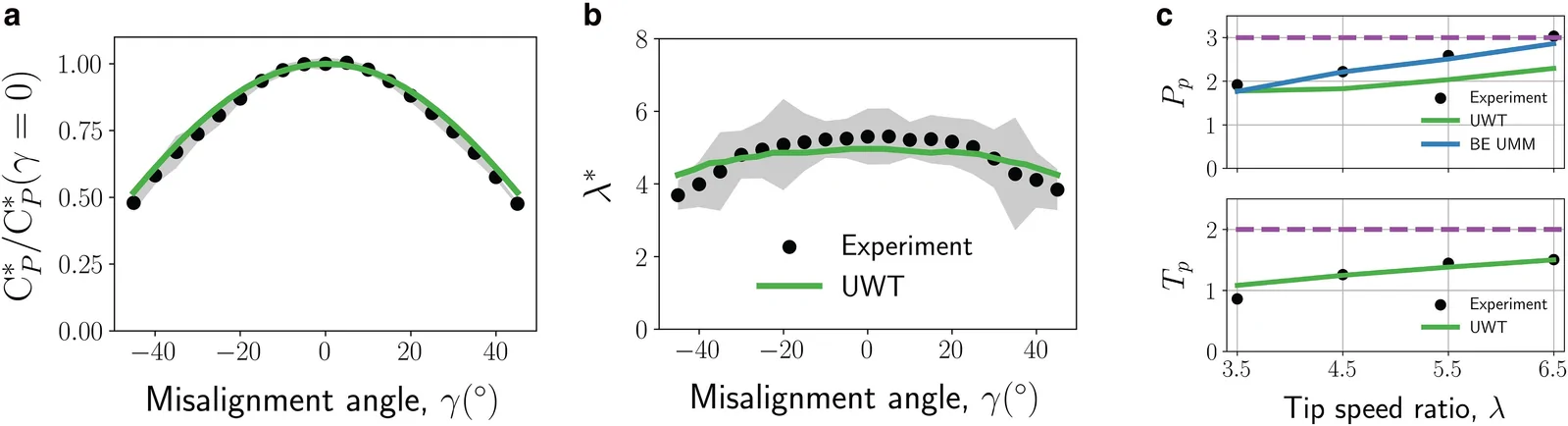
The UWT model predictions of the power maximizing control strategy at each yaw misalignment angle compared to the experiments. a) The maximum coefficient of power at each yaw angle $C_{P}^{*}$, evaluated at the optimal tip speed ratio $\lambda^{*}$. b) The power maximizing tip speed ratio $\lambda^{*}$ depending on the yaw angle (shading indicates variation obtained by removing tangential induction submodel). c) Power-yaw exponent using the form $C_{P}/C_{P}(\gamma =0)=\cos^{P_{p}}\;(\gamma )$ fit to the experiments and model results using least-squares regression. A standard value of $P_{p}=3$ is shown for reference, which is the loss anticipated by geometry. Thrust-yaw exponent $T_{p}$ is also shown, following $C_{T}/C_{T}(\gamma =0)=\cos^{T_{p}}\;(\gamma )$. A standard value of $T_{p}=2$ is shown for reference, which is the loss anticipated by geometry.

Finally, the UWT model is further able to predict the dependence of the thrust-yaw and power-yaw relationships on the tip speed ratio, as demonstrated by comparing the best-fit $T_{p}$ and $P_{p}$ exponents from the experiments and model in Fig. 3c. We consider tip speed ratios that yield <25% deviation from $C_{P}^{*}$ in yaw aligned operation to focus on regimes where aerodynamic losses are lower. The experimental results shown in Fig. 3c demonstrate that empirical cosine modeling cannot be generalized across control strategies, since $T_{p}$ and $P_{p}$ depend on the tip speed ratio. Together, these results demonstrate that the UWT model can predict the complex dependence of wind turbine power and thrust on multiple control variables simultaneously. The robust ability demonstrated here of the UWT model to predict the complex aerodynamics of a wind turbine across operational strategies as well as the power maximizing tip speed ratio for all misalignment angles unlocks its use in wind farm flow control optimization at scale.

## Discussion and UWT model implications

There are major uncertainties in the aerodynamic models that are currently used to guide wind farm design and flow control methods such as wake steering (9, 12). Given variations in wind turbine designs, wind farm layouts, meteorological conditions, and geographical factors, the power maximizing control strategy will be different at each wind farm and will also depend dynamically on atmospheric conditions (20). Because collective flow control is a high-dimensional optimization problem, it is currently intractable to use high-fidelity modeling methods based on computational fluid dynamics within control workflows. Thus, fast-running aerodynamic models are essential to wind farm planning and operation (2, 12), so that site-specific decisions can be customized at low-cost and with speed. However, prior to their use, these models must be designed and validated using high-fidelity experimental data, produced in a controlled setting at flow conditions relevant to full-scale wind turbine operation. Beyond flow control, predicting and reducing the power lost by unintended yaw misalignment can provide significant value to wind turbines (15, 66, 67). Critical uncertainties have remained for the power and loads on a wind turbine across control states (i.e. yaw misalignments and tip speed ratios) because of the lack of data collected at dynamic similarity without confounding, spatiotemporally varying atmospheric effects (9).

The UWT model, validated in the previous section, offers several key opportunities to improve wind farm flow control. First, the UWT model can be used to predict the power production and initial wake velocities for arbitrary input of yaw misalignment, blade pitch, and tip speed ratio. Existing wake steering controllers that leverage cosine models for thrust and power are strongly dependent on the tuned empirical parameters. The optimized setpoints for yaw misalignment depend on these parameters (18). But these parameters are not known a priori without costly simulations (18) or field tests that could be months in length (9), and incorrectly estimating the empirical parameters can lead to significant performance degradation (68). To reduce uncertainty for wake steering flow control, the UWT model offers a new method to predict how wind turbine thrust and power depend on the yaw misalignment, blade pitch, and tip speed ratio, without relying on uncertain empirical parameters. Second, the results in Fig. 3b indicate that the tip speed ratio that maximizes wind turbine power depends on the yaw misalignment angle. This offers new insight into a generalized flow control approach that combines wake steering (yaw misalignment) with thrust control (changes to pitch and tip speed ratio).

To evaluate the impacts of the UWT model on wind farm flow control, we conduct a numerical case study of a large wind farm in Wisconsin, United States, similar to the national-scale wake steering evaluation presented by Simley et al. (69). The wind farm of interest is shown in Fig. 4a. The UWT model is coupled with a wake model to predict wind farm flow and power production. Following Simley et al. (69), we use the Gauss–Curl wake model (70). The technical details of the wake modeling, coupling to the UWT model, and other aspects of the setup are provided in the SI Appendix. Modeling turbulent wakes associated with wind turbines continues to represent a major challenge due to the multiscale flow physics and high-dimensional dependencies inherent to the problem (48, 71). As such, there are substantial uncertainties associated with the specific quantitative estimates of the potential for wind farm flow control derived from case study demonstrations that are based on engineering wind farm flow models, such as the Gauss–Curl model presently used. The present results are intended for illustrative purposes to highlight the potential associated with wind farm flow control based on the proposed UWT and the associated uncertainties are in line with recent case studies (21, 72). Using standard greedy control where each turbine is controlled individually to maximize its own power (Fig. 4c), the model estimates that the wake losses in annual energy production (AEP) at this wind farm are 10.2% which, given standard power purchase agreement prices, could result in millions of dollars of wake losses per year.

**Figure 4:**
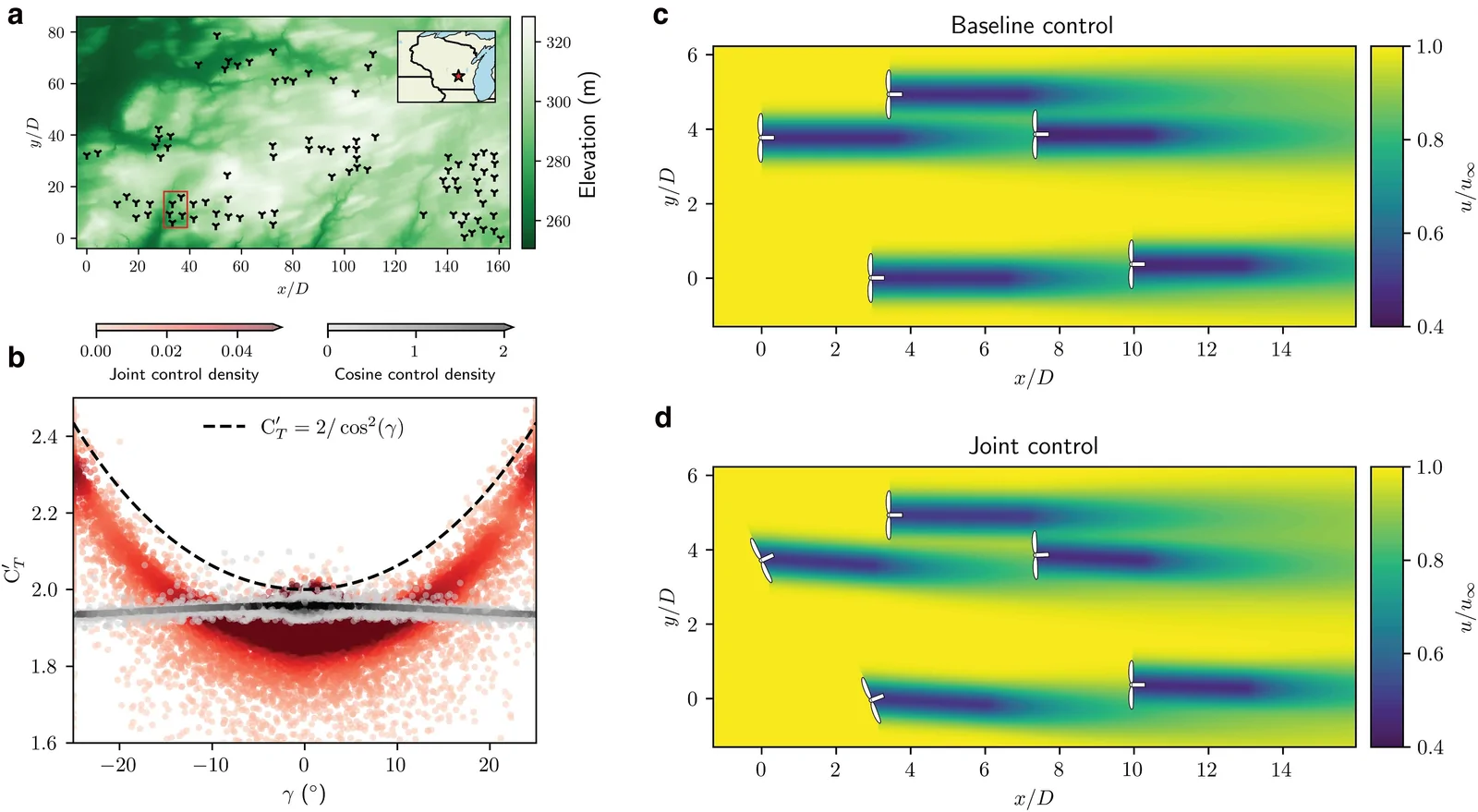
Wind farm flow control case study. a) Map of the wind farm of interest with 90 Vestas 1.8 MW turbines with the coordinates normalized by the rotor diameter *D*. b) Optimized wind farm flow control setpoints for each of the 90 turbines across all wind speeds and directions for the baseline control method that uses wake steering (yaw misalignment) based on a cosine turbine model and also for joint wake steering and thrust control using the UWT model developed and validated here. Setpoints are the yaw misalignment *γ* and the local thrust coefficient $C_{T}^{\prime }$, where the thrust force is proportional to $C_{T}^{\prime }$ times the squared velocity at the rotor. The theoretical estimate for the power maximizing local thrust coefficient $C_{T}^{\prime }$ for an individual turbine in yaw (28), $C_{T}^{\prime }=2/\cos^{2}\;(\gamma )$ is shown as a reference. c) Wind flow predicted by an engineering wake model for a subset of the wind farm in baseline greedy control. d) Wind speed, normalized by the freestream, predicted by an engineering wake model for a subset of the wind farm in optimized joint wake steering and thrust flow control.

To represent current wake steering flow control methods, we first represent the rotor thrust and power using cosine models, as described in the previous section. To simulate an optimistic setting where the thrust and power dependence on the yaw angle is known perfectly, we fit $T_{p}$ and $P_{p}$ to the UWT model thrust and power, respectively, as a function of the yaw angle and assume the pitch angle is fixed at the yaw-aligned value with torque controlled following a standard $k-\Omega^{2}$ protocol (9). This yields empirical cosine model exponents $T_{p}=1.03$ and $P_{p}=1.53$, with which we perform wake steering optimization by identifying the yaw misalignment setpoints that maximize power based on the incident wind speed and direction to the farm. Applying those optimized yaw angles to the model wind farm represented by the UWT model yields estimated gains in AEP of 1.68% through yaw control. However, prior to improved modeling presented in this study, the cosine exponents would be unknown and standard geometric values of $T_{p}=2$ and $P_{p}=3$ must be used. Then, the AEP gain from the resulting yaw angles optimized with these faulty parameters would be only 1.27%, a reduction in the energy gain of ≈25%. Finally, using the UWT to perform synergistic optimization of yaw, tip speed ratio, and pitch angles, results in an AEP gain of 1.77%. The resulting setpoints for yaw and thrust comparing the joint control based on the UWT model and wake steering control based on the faulty cosine model are shown in Fig. 4b, and the corresponding flow field is shown in Fig. 4d. Assuming a constant power purchase agreement price of $50/MWh (73), the joint control based on the UWT model yields a gain in revenue of $11,125 per turbine per year. Together, the UWT model increases the AEP and revenue gain compared to wake steering with inaccurate rotor modeling, represented by faulty cosine exponents, by up to 40%. While there remain uncertainties associated with these case study estimates stemming from the flow physics and wind plant modeling, the results shown here illustrate the potential opportunity for UWT to improve wind farm flow control. This potential should be evaluated in future field experiments at full-scale wind farms. Moving forward, similar wind farm flow optimization based on the validated UWT could consider other objectives, such as reducing the land or water area footprint of wind farms (21) or minimizing loading within wind farms to extend turbine lifetime.

The opportunity to increase wind farm flow control performance and the power capture of unintentionally yaw misaligned wind turbines using the UWT model is enabled by the experimental validation at full dynamic similarity. More broadly, the full dynamic similarity experimental testbed developed here unlocks the ability to explore unanswered research questions for high Reynolds number aerodynamics of turbines and other rotors in controlled tests that isolate key flow physics. As wind turbines grow in hub-height and rotor diameter, the increasing length scales and complex inflows experienced have pushed the aerodynamics of these machines into unchartered territories. Critical new questions are emerging at the forefront of flow-structure interactions, aeroelasticity, vibrations, unsteadiness, flow control, and turbulent wake dynamics (2, 3, 12), but controlled experimental data at field-relevant Reynolds numbers for these applications are scarce or absent. Expanding field-relevant data to validate physical models reduces uncertainty and could lead to efficiency gains and cost reductions. Through pressurization, full dynamic similarity may be achieved, stimulating a new generation of experimental data, validation, and flow physics insights to spark innovations across wind power and rotor dynamics applications.

## Materials and methods

### Wind tunnel experiments

Experiments are performed at a Reynolds number of four million in the Princeton HRTF. The velocity fluctuations along the centerline are below 1.2% and have an integral length of 0.46D. The flow across the 49 cm-diameter circular cross section of the wind tunnel is uniform to within 4% (57). The relatively low fluctuations and high uniformity are achieved using honeycomb and wire mesh screens placed upstream of the turbine test section. Static pressure in the tunnel loop is measured using a pressure transducer (Omega PX419, range: ±24 MPa, accuracy: ±0.08%). Tunnel temperature is measured using a resistance temperature detector (Omega PR-10, accuracy: ±0.20° C). These measurements are used to estimate the fluid density and viscosity using the method outlined in Ref.(74). A pitot-static tube placed 5.1D upstream of the turbine connected to a pressure transducer (Validyne DP15, range: 13.8 kPa, accuracy: ±69 Pa) measures the dynamic pressure ($\rho {U_{\infty }}^{2}/2$) of the flow, from which the freestream velocity is calculated. In our experiments, the air density is increased to 130 kg m${\;}^{-3}$ via facility pressurization at a flow velocity of 4.35 ms${\;}^{-1}$. Crucially, the capability to independently control density and velocity in the HRTF decouples the Reynolds number ReD and the tip speed ratio *λ* while avoiding compressible flow effects, a feat that is unachievable in conventional wind tunnels which control velocity alone.

A 15 cm-diameter, self-starting, and free-spinning wind turbine model is mounted in the test section of the HRTF (Fig. 1b), resulting in a solid blockage of 9.4% when yaw-aligned. With this rotor at the aforementioned facility conditions, an operational Reynolds number of ReD=4×106 is reached, significantly higher than previous studies that consider yaw misalignment. The three rotor blades are designed using NACA 63 series airfoil profiles with radial chord length, thickness, and pitch schedules suited to the operational Reynolds number employed, yielding a rotor with performance typical of modern commercial turbines, $C_{P}=[0.4,0.5]$ and $C_{T}=[0.7,0.9]$ for λ=[3,8] (75). The detailed rotor geometry is described in the SI Appendix. The rotor is connected to the turbine tower with a gearbox housed in the nacelle that transfers power to a torque transducer (Magtrol TM-305, range: ±2 N m, accuracy: ±0.003 N m) to facilitate torque and rotational speed measurements. The turbine tower is mounted to a load cell (JR3 Inc. 75E20A4, range: ±200 N, accuracy: ±0.5 N) to record forces in all three component directions. The turbine thrust force is resolved from the total force measured in the rotor-normal direction by subtracting the drag force measured in the same direction and flow conditions without the rotor installed. As the turbine is free-spinning, the rotational speed is controlled by an electromagnetic hysteresis brake (Magtrol AHB-3) independent of the freestream velocity, with tests conducted at λ= 3.5, 4.5, 5.5, 6.5, and 7.5. Yaw misalignment is introduced by rotating the turbine between −45${\;}^{\circ }$ and 45${\;}^{\circ }$, with 0 ° corresponding to the rotor rotation axis being aligned with the freestream flow direction with the rotor blades facing the incoming flow (Fig. 1c). The turbine is swept through the full range of *γ* angles, while maintaining *λ* by adjusting the brake level. Force, torque, and rotor speed are collected at each test case for 30s to achieve statistically converged measurements. For each *γ*, a polynomial fit is applied to the *λ*-$C_{P}$ curve to obtain an optimal *λ* value, $\lambda^{*}$, which maximizes $C_{P}$. Measurements are repeated at each *γ* with $\lambda^{*}$ to obtain power-optimal $C_{T}$ and $C_{P}$, denoted $C_{T}^{*}$ and $C_{P}^{*}$, respectively. A full quantification of the experimental uncertainties is provided in the SI Appendix.

### Wind turbine modeling

The wind turbine rotor is modeled using the Unified Momentum Model (63), which predicts the induction of rotors under arbitrary thrust coefficients and misalignment angles between inflow and the rotor. The model addresses two limitations of classical momentum theory. First, classical momentum theory fails at higher thrust coefficients, usually around values of the induction factor $a_{n}=1-{\vec{u}}_{d}\cdot \hat{n}/(U_{\infty }\cos\;(\gamma ))\approx 0.37$ (76), where ${\vec{u}}_{d}$ is the velocity at the rotor, $\hat{n}$ is the rotor-normal unit vector, $U_{\infty }$ is the freestream wind speed, and *γ* is the rotor misalignment angle. This limitation is addressed in the Unified Momentum Model by predicting the static pressure in the wake, which remains persistently lower than the freestream pressure at high thrust coefficients (63, 77). Second, classical momentum theory is 1D, and cannot predict the effects of misalignment without empirical corrections (26). To address this limitation, the Unified Momentum Model predicts the lateral velocity generated by misalignment using a lifting line approach (78) and accounts for the lateral velocity in mass, momentum, and energy balances (28). Together, the Unified Momentum Model is represented by a coupled set of analytical equations that can be solved efficiently using fixed-point iteration (see SI Appendix). In this study, we utilize an extension of the Unified Momentum Model that accounts for geometrical blockage (64) on the rotor aerodynamics, with full information provided in the SI Appendix. For clarity and brevity, this extended model will hereafter be referred to as the Unified Momentum Model. As described in the wind tunnel experimental setup, for the 15.0 cm diameter rotor with a 49.0 cm tunnel interior diameter, the blockage ratio in yaw alignment is 9.4%.

The Unified Momentum Model is the generalization of classical momentum theory that accounts for arbitrary operational conditions (thrust coefficient and misalignment angle). Momentum theory uses an actuator disk approach to predict induction and power of wind turbine rotors based on an input thrust coefficient (58), but the thrust coefficient is an unknown input to the model. To predict the thrust coefficient, the standard solution is to use a blade element model that predicts the lift and drag forces on the rotating wind turbine blades (airfoils) based on the velocity at the rotor (29, 79), accounting for induction at the rotor using momentum theory. We couple the Unified Momentum Model to a blade element model, resulting in the UWT model that predicts wind turbine forces and power across all relevant operational regimes (blade pitch angle, tip speed ratio, and rotor misalignment angle) without using high thrust (80) or skewed wake corrections (81) that are ubiquitous in wind power modeling (26, 82). The coupling between the Unified Momentum Model and the blade element model is described by [$a_{n},u_{4},v_{4}]=f_{\mathrm{Unified}}(C_{T},\gamma )$, where the outputs of the Unified Momentum Model $f_{\mathrm{Unified}}$ are $a_{n}$, the induction, $u_{4}$, the streamwise wake velocity, and $v_{4}$, the lateral wake velocity from misalignment. The input is the thrust coefficient $C_{T}$ that is predicted by the blade element model (full equations provided in the SI Appendix) and the rotor misalignment angle *γ*. Furthermore, to assess the accuracy of the Unified Momentum Model without the error incurred by solving the coupled BEM equations, we compute $a_{n}$ directly from the experimentally measured $C_{T}$ and compute the turbine power using the airfoil forces predicted by the blade element model (BE UMM) in Fig. 2c. For reference, the power-yaw relationship from actuator disk only (AD UMM), $P_{\mathrm{AD}}={\vec{F}}_{T}\cdot {\vec{u}}_{d}$ is also plotted.

## Supplementary Material

pgag303_Supplementary_Data

## Data Availability

The data described in this study will be published open-access upon the publication of the manuscript. The code used in this study is open-source. The Unified Wind Turbine (UWT) is open-source and implemented in MITRotor https://github.com/Howland-Lab/MITRotor. The wind farm modeling is open-source and implemented in MITWindfarm https://github.com/Howland-Lab/MITWindfarm.
