# Supplementary material for: Full dynamic similarity experiments and predictive modeling of wind turbine aerodynamics across control strategies
Source: PNAS Nexus. 2026 Sep 7;5(10):pgag303. doi: 10.1093/pnasnexus/pgag303 (PMC13626229; doi:10.1093/pnasnexus/pgag303)
Supplement: Supplementary file 1 [file pgag303_supplementary_data.zip › Kurelek_etal_PNAS_Nexus_2026_SI_Appendix.pdf]

Supporting Information for

Full dynamic similarity experiments and predictive modeling of wind turbine aerodynamics across control strategies

John W. Kurelek, Ilan M. L. Upfal, Supun Pieris, Kirby S. Heck, Alexander Piqué, Marcus Hultmark, & Michael F. Howland

John W. Kurelek, Michael F. Howland.
E-mails : john.kurelek@queensu.ca, mhowland@mit.edu

This PDF file includes:

Supporting text
Figs. S1 to S8
Tables S1 to S2
Legends for Dataset S1 to S2
SI References

Other supporting materials for this manuscript include the following:

Datasets S1 to S2

Supporting Information Text

Materials and Methods

Wind turbine rotor geometry. A single blade of the three-bladed rotor and associated geometrical parameters are shown in Fig. S1. The wind turbine rotor is manufactured from a single block of 7075 Aluminum alloy using computer numerical control milling. An aerodynamically smooth surface is ensured by finishing to a root-mean-square roughness height, 0.2 µm, that is an order of magnitude lower than the critical roughness height, 8–30 µm, estimated by Miller *et al.* (1) for the same model and operating conditions. The nosecone is a NACA 1 series spinner appropriately scaled for the rotor diameter. The blades are designed in two sections, with the blade angle consisting of a fixed and variable component, $\theta = \theta_p + \theta_t(r)$, where θ_p is a fixed pitch angle of 5° and $\theta_t(r)$ is a bipartite variable twist schedule (Fig. S1d). In the first section, starting from the rotor hub, the twist is 13° and the chord and thickness are varied to transition from a circular profile to a NACA 63-235 profile at 14.8% of the blade radius. In the second section, the chord, thickness and twist are reduced with increasing radial position, transitioning to a NACA 63-214 profile with zero twist at the tip ($\theta_t(r = R) = 0$). The axis of the blade angle is located at the quarter chord position, except at the hub where it coincides with the center of the circle.

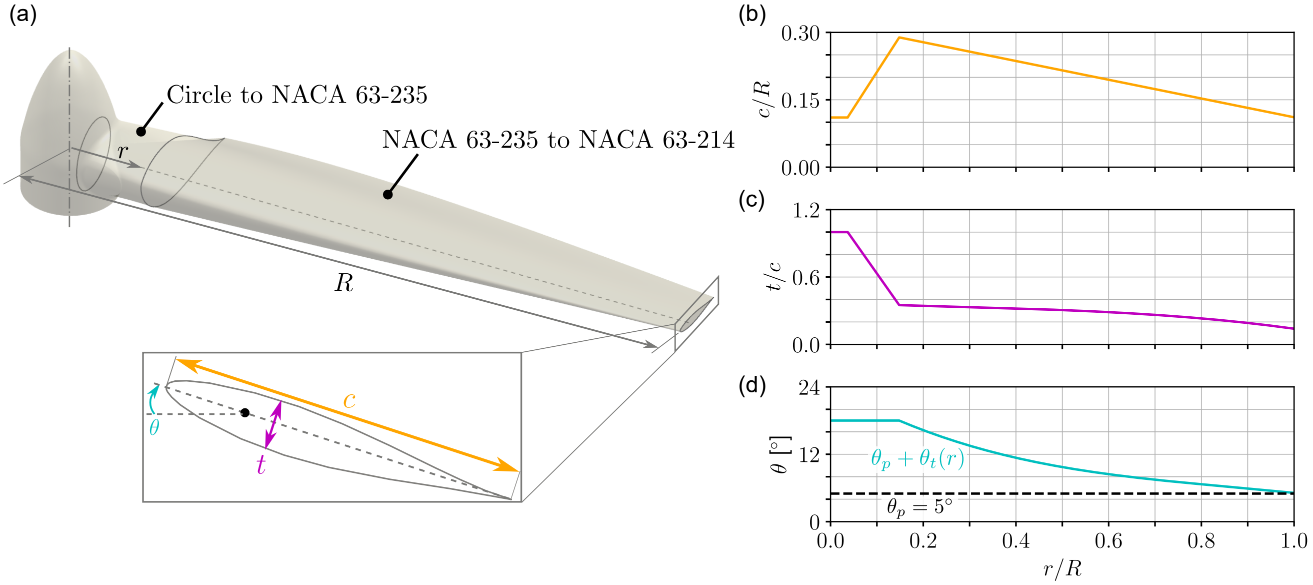

Fig. S1. Rotor blade geometry. (a) Schematic of the wind turbine rotor blade showing the various blended geometries from hub to tip. (d,e,f) Quantitative description of the rotor blade along the radial coordinate normalized by rotor blade radius, r/R, showing normalized sectional chord length, c/R, thickness, t/c, and blade angle, θ, composed of a 5° fixed pitch (black dashed line) and a variable twist schedule.

Experimental uncertainty. Estimation of experimental uncertainty of λ, C_T, and C_P is presented in this section. All quantities are determined on a 95% confidence interval and combined when required using a root-sum-square (RSS) operation according to the nth order uncertainty methodology (2). Uncertainty of a quantity a is denoted ϵ_a.

Sensor uncertainty. The full set of experimental measurements includes sensor data collected from the tunnel static pressure transducer, tunnel temperature sensor, pitot-static pressure transducer, load cell, and torque transducer. For all measurements, the sensor resolution uncertainty dominates over the resolution of the data acquisition hardware. The uncertainties associated with each of these sensors stemming from sensor resolution and calibration are summarized in Table S1.

Table S1. Summary of sensor uncertainties

Parameter	Symbol	Uncertainty	Unit
Tunnel static pressure	P_∞	±19200	Pa
Tunnel temperature	T_∞	±0.2	°C
Pitot-static pressure	P_{dyn}	±69	Pa
Thrust	F_T	±0.5	N
Torque	τ	±0.0005	N m
Yaw	γ	±0.1	°

Higher order uncertainties. Apart from sensor uncertainties, there are a number of other uncertainty sources originating from the experimental and data processing methodologies. There are two significant temporal uncertainty components: drift and fluctuations. Signal drift occurs for all sensors due to variation of the tunnel temperature, which is most significant when operating at relatively high static pressures. Sensor drift is minimized and a consistent level of uncertainty is maintained by limiting all tests to a maximum temperature change of 1 °C or less assessed through pre- and post-experiment measurements conducted under quiescent conditions. Below this threshold, the contribution of sensor drift to uncertainty is quantified by the rise observed in the time histories of each signal according to a fitted linear trend line. The contribution of temporal fluctuations to total uncertainty is estimated by first de-trending the data according to the estimated temperature drift, after which twice the standard error of the mean is calculated and used to quantify temporal uncertainty on a 95% confidence interval. Temporal uncertainties are incorporated with other sources using the RSS method.

Uncertainties of directly measured quantities are propagated to derived quantities in two ways. For a derived quantity Y that can be explicitly defined as a function of other measured or derived quantities X_i, as $Y = f(X_i)$, uncertainty ϵ_Y, is estimated according to:

$$\epsilon_Y = \sqrt{\sum_{i=1}^{N} \left(\frac{\partial Y}{\partial X_i} \epsilon_{X_i} \right)^2}. \qquad [1]$$

This method is employed when the function f is known and evaluation of its partial derivatives is straightforward. When this is not possible, *e.g.,* when using advanced multi-step analysis techniques, the method of sequential perturbation (MSP) (3) is used instead.

A summary of uncertainty estimates of all quantities, measured and derived, is presented in Table S2. Propagation of uncertainty, using either Eq. (1) or MSP, is used for all derived quantities except two in particular. First is the rotor rotational speed, Ω, as the sensor uncertainty is not provided by the manufacturer. Second is the freestream velocity, derived from dynamic pressure measured via the freestream pitot-static tube and accompanying pressure transducer (*cf.* Table S1). This sensor must have a large sensing range to accommodate the vast operational envelope of the HRTF ($1 \leq U_\infty \leq 10\,\mathrm{m\,s^{-1}}$, $0.1 \leq P_\infty \leq 24\,\mathrm{MPa}$), leading to relatively high uncertainties at the operating conditions of this investigation (*e.g.*, $\epsilon_{P_{\mathrm{dyn}}} = \pm 69\,\mathrm{Pa}$ yields at least $\epsilon_{U_\infty} = \pm 6\%$ for $U_\infty = 4.35\,\mathrm{m\,s^{-1}}$ and $P_\infty = 11.4\,\mathrm{MPa}$). This is only of significant consequence to the accurate resolution of instantaneous values of U_∞ and associated derived properties, while the presented results are ensemble-averaged values which should statistically represent the effect of the number of samples (*i.e.*, more samples should decrease uncertainty). A similar argument can also be made for estimates of Ω. As such, uncertainty in Ω and U_∞ is quantified using only the standard error of the mean (SEM) approach, which is reported in Table S2.

Table S2. Summary of uncertainties accounting for higher order sources

Parameter	Symbol	Method	Uncertainty	Unit
Density	ρ	MSP	1	$\mathrm{kg\,m^{-3}}$
Kinematic viscosity	ν	MSP	10^{-9}	$\mathrm{m^2\,s^{-1}}$
Freestream velocity	U_∞	SEM	10^{-4}	$\mathrm{m\,s^{-1}}$
Thrust	F_x	RSS	1.5	N
Torque	τ	RSS	0.0014	N m
Rotor speed	Ω	SEM	7.3	$\mathrm{rad\,s^{-1}}$
Reynolds number	Re_D	Eq. (1)	14000	-
Tip-speed ratio	λ	Eq. (1)	Fig. S2	-
Thrust coefficient	C_T	Eq. (1)	Fig. S2	-
Power coefficient	C_P	Eq. (1)	Fig. S2	-

Relative uncertainties of λ, C_T, and C_P for each λ level tested are presented in Fig. S2. Notably, the uncertainty of λ is below 2% for nearly all cases due to high measurement accuracy and low temporal variation of rotor speed. The relative uncertainty of C_T is between 6% - 10%, while for C_P the uncertainty is less than 4%. The higher relative uncertainty in C_T relative to C_P stems from sensor resolution. Notably both C_T and C_P relative uncertainties increase due to decreased performance with increasing yaw misalignment, while the sensor uncertainty remains constant. The increasing relative uncertainty with yaw misalignment is more pronounced for C_T due to the higher absolute uncertainty relative to C_P.

Uncertainty of λ^****,*** C_T^****, and*** C_P^****.*** During measurements, selection of λ^* is based on maximizing C_T and C_P from the first round of tests at each γ for $\lambda =$ 3.5, 4.5, 5.5, 6.5, and 7.5. A polynomial fit to $\lambda - \mathrm{C}_T$ and $\lambda - \mathrm{C}_P$ is used to estimate the optimal operating point that maximizes turbine performance. Measurements are then conducted at this optimal operation point and adjustments are made in-situ if the turbine performance is below the predicted level.

Slight disparate estimates of the optimal operating point result based on either maximizing C_T or C_P, introducing a methodological uncertainty in addition to measurement uncertainty for λ^*, C_T^*, and C_P^*. This methodological uncertainty on λ^* is quantified by the difference between the λ^* setpoint, and the predicted λ^* from either maximizing C_T or C_P, resulting in two methodological uncertainty contributions to λ^*. These two sets of uncertainties are averaged, then combined with the

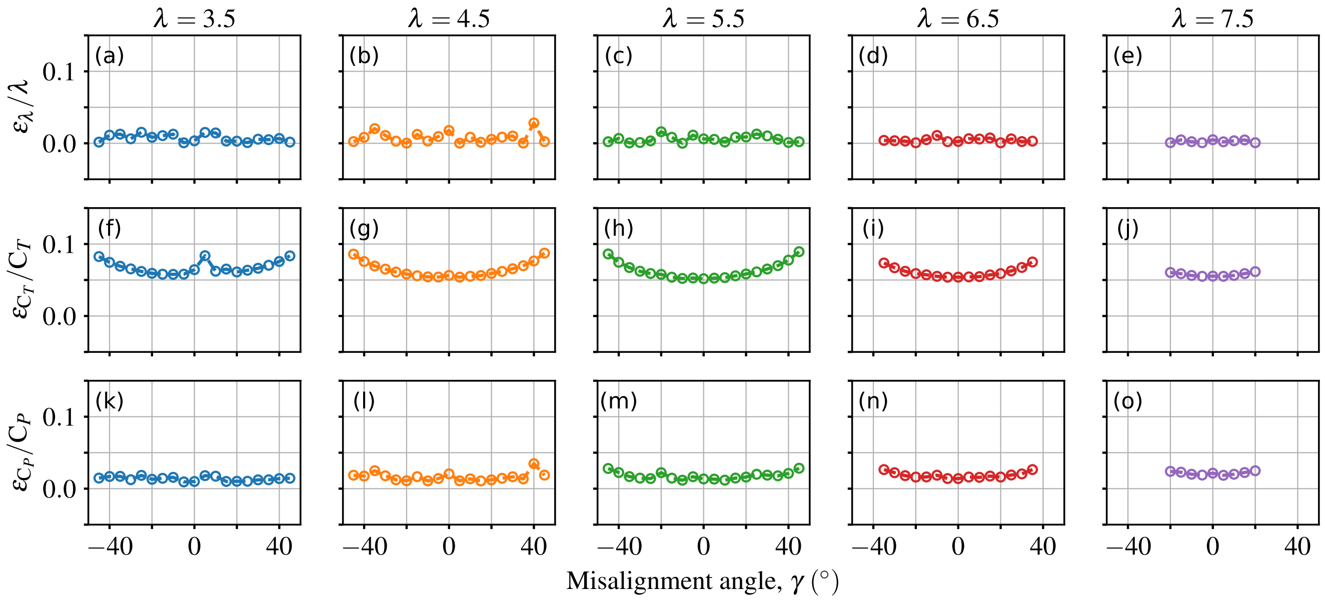

Fig. S2. Relative experimental uncertainties of ensemble-averaged turbine operating parameters as a function of yaw-misalignment. All uncertainties are on a 95% confidence interval and incorporate sensor and measurement uncertainty sources. (a-e) Tip-speed ratio. (f-j) Thrust coefficient. (k-o) Power coefficient at the investigated tip-speed ratio setpoints (columns).

measurement uncertainty using a root-sum-square operation to estimate the final ϵ_{λ^*}. A similar procedure is undertaken for $\epsilon_{C_T^*}$ and $\epsilon_{C_P^*}$ to estimate final uncertainties that account for selection of the optimal operating point.

Uncertainties of λ^*, C_T^*, and C_P^* accounting for both measurement and methodological contributions are presented in Fig. S3. Most notably, ϵ_{λ^*} is significantly higher compared to the fixed setpoint cases (Fig. S2), mainly due to the optimal operating point selection process. In contrast, $\epsilon_{C_T^*}$ and $\epsilon_{C_P^*}$ are comparable to ϵ_{C_T} and ϵ_{C_P} due to the relatively low sensitivity of C_T and C_P to λ close to λ^*.

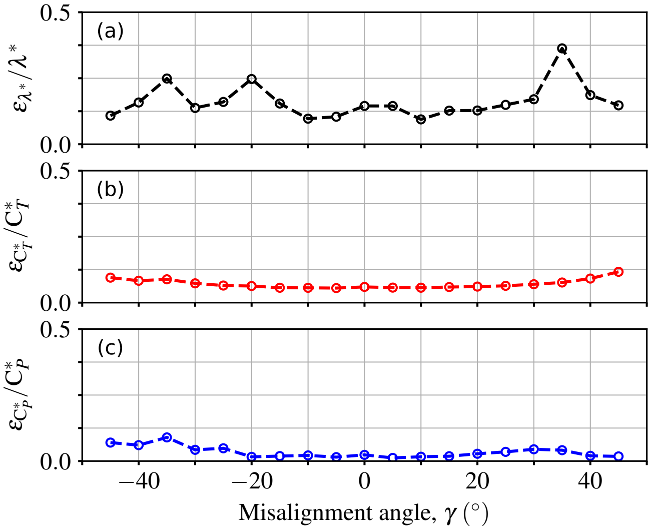

Fig. S3. Relative experimental uncertainties with respect to yaw-misalignment of ensemble-averaged turbine operating parameters under optimal operating conditions. Uncertainties represent a 95% confidence interval and incorporate sensor and measurement uncertainty sources, along with methodological uncertainty of optimal operating point selection. (a) Tip-speed ratio. (b) Thrust coefficient. (c) Power coefficient.

A Unified Wind Turbine model. The underlying equations of the Unified Wind Turbine model, which couples the Unified Momentum Model with blade element modeling, are presented in this section. The model is available open-source on GitHub (https://github.com/Howland-Lab/MITRotor).

Unified Momentum Model. The Unified Momentum Model (4) solves for the induction a_n, streamwise wake velocity u_4, lateral wake velocity v_4, near-wake length x_0, and the wake pressure deficit $p_4 - p_1$ using the following coupled equations

$$a_n = 1 - \sqrt{\frac{u_\infty^2 - u_4^2 - v_4^2}{C_T' \cos^2(\gamma) u_\infty^2} - \frac{(p_4 - p_1)}{\frac{1}{2}\rho C_T' \cos^2(\gamma) u_\infty^2}}, \quad [2]$$

$$u_4 = -\frac{1}{4} C_T'(1-a_n)\cos^2(\gamma) u_\infty + \frac{u_\infty}{2} + \frac{1}{2}\sqrt{\left(\frac{1}{2}C_T'(1-a_n)\cos^2(\gamma)u_\infty - u_\infty\right)^2 - \frac{4(p_4-p_1)}{\rho}}, \quad [3]$$

$$v_4 = -\frac{1}{4}C_T'(1-a_n)^2 \sin(\gamma)\cos^2(\gamma) u_\infty, \quad [4]$$

$$\frac{x_0}{D} = \frac{\cos(\gamma)}{2\beta}\frac{u_\infty + u_4}{|u_\infty - u_4|}\sqrt{\frac{(1-a_n)\cos(\gamma)u_\infty}{u_\infty + u_4}}, \quad [5]$$

$$p_4 - p_1 = -\frac{1}{2\pi}\rho C_T'(1-a_n)^2\cos^2(\gamma)u_\infty^2 \arctan\left[\frac{1}{2}\frac{D}{x_0}\right] + p^{NL}(C_T', \gamma, a_n, x_0). \quad [6]$$

The rotor-normal, rotor-averaged induction is defined as

$$a_n = 1 - \frac{\vec{u}_d \cdot \hat{n}}{u_\infty \cos(\gamma)}, \quad [7]$$

which is the generalization of the standard (axial) induction factor in classical one-dimensional momentum modeling (5). The freestream wind speed is u_∞, the density is ρ, and the rotor diameter is D. The only parameter is the near wake spreading rate, which is set to a constant value of $\beta = 0.1403$, consistent with shear layer turbulence literature (4). The near wake length is x_0. The prognostic equation for pressure (Eq. (6)) contains two terms. The first term is from actuator disk forcing. The second term (p^{NL}) is a nonlinear term that results from advection.

If C_T is used as the input thrust coefficient variable instead of C_T', a sixth equation is included in the set of equations above:

$$C_T' = \frac{C_T}{(1-a_n)^2\cos^2(\gamma)}. \quad [8]$$

The thrust coefficient is defined as $C_T = 2\|\vec{F}_T\|/(\rho A_d u_\infty^2)$, where $\|\vec{F}_T\|$ is the magnitude of the thrust force and $A_d = \pi D^2/4$ is the rotor swept area.

In this study, an extension of the Unified Momentum Model is used which accounts for solid blockage, described in Upfal *et al.* (2026) (6). Solid blockage is defined as the ratio between the rotor swept area and the wind tunnel cross-section (7). As a consequence of blockage, the static pressure in the flow differs from an unblocked environment as the flow around the turbine must accelerate relative to the freestream flow to conserve mass along with the wake velocity deficit (8, 9). This effect violates the assumption in the Unified Momentum Model (4) that the pressure outside the streamtube containing the turbine and its wake is equal to the freestream pressure. In the next section, we describe the extension of the Unified Momentum Model to account for blockage effects. In the main text, the model that accounts for blockage is used for all model predictions.

Extension of the Unified Momentum Model to account for blockage. The generalization of the Unified Momentum Model to rotors operating in confined flows (blockage) is given by the following system of six coupled nonlinear equations,

$$a_n = 1 - \sqrt{\frac{u_\infty^2 - u_4^2 - v_4^2}{C_T'\cos^2(\gamma)u_\infty^2} + \frac{(p_1 - p_{4,w})}{\frac{1}{2}\rho C_T'\cos^2(\gamma)u_\infty^2}} \quad [9]$$

$$\frac{u_4}{u_\infty} = \frac{A_d}{A_4}(1-a_n)\cos(\gamma) \quad [10]$$

$$\frac{v_4}{u_\infty} = -\frac{1}{4}C_T'(1-a_n)^2\sin(\gamma)\cos^2(\gamma) \quad [11]$$

$$\frac{u_s}{u_\infty} = 1 + \frac{\beta A_4(u_\infty - u_4)}{(A_d - \beta A_4)u_\infty} \quad [12]$$

$$\frac{A_4}{A_d} = \frac{\frac{1}{2}C_T'(1-a_n)^2\cos^3(\gamma)u_\infty^2 + (1/\beta)(u_s^2 - u_\infty^2 - (p_1 - p_4)/\rho)}{(p_4 - p_{4,w})/\rho + u_s^2 - u_4^2} \quad [13]$$

$$\frac{p_1 - p_4}{\rho u_\infty^2} = \frac{1}{2}(u_s^2/u_\infty^2 - 1) \quad [14]$$

where the freestream incident flow speed is u_∞, the fluid density is ρ, the actuator disk area is A_d, and $\beta = A_d/A_{CV}$ is the blockage ratio defined using the control volume (channel) cross section, A_{CV}. The bypass velocity is u_s, the outlet wake area is A_4, the inlet pressure is p_1, the outlet pressure in the bypass flow is p_4, and the outlet pressure within the wake is $p_{4,w}$. The pressure difference between the wake and bypass flow is $p_{4,w} - p_4 = (1-\beta)P_{\mathrm{UMM}}(C_T', \gamma)$ where P_{UMM} is the base suction pressure deficit (Eq. 6) given by the Unified Momentum Model (4). If C_T is used as the input thrust coefficient variable instead of C_T', an additional equation is included in the set of equations above:

$$C_T' = \frac{C_T}{(1-a_n)^2\cos^2(\gamma)}. \qquad [15]$$

The thrust coefficient is defined as $C_T = 2\|\vec{F}_T\|/(\rho A_d u_\infty^2)$, where $\|\vec{F}_T\|$ is the magnitude of the thrust force. In the main text, the converged solution to these equations (Eqs. 9-15) is compactly written as $[a_n, u_4, v_4] = f_{\text{Unified}}(C_T, \gamma)$. A full derivation and validation of this model is presented in Upfal *et al.* (2026) (6).

Blade element modeling. The blade element model is formulated in non-dimensional radial units, $\mu = r/R$, where $R = D/2$ is the rotor radius. The inputs are the rotor misalignment angle γ, blade pitch angle θ_p, and tip speed ratio $\lambda = \Omega R/U_\infty$. The turbine model is characterized by blade solidity $\sigma(\mu) = Bc(\mu)/(2\pi R\mu)$ (with the number of turbine blades B and chord length $c(\mu)$), blade twist angle, $\theta_t(\mu)$, and airfoil lift and drag curves $C_l(\mu,\alpha)$ and $C_d(\mu,\alpha)$, which depend on radial position μ and angle of attack α. The blade element equations are solved on a polar grid of (μ,ψ), where ψ is the azimuthal position. The local freestream non-dimensional wind speed is $U(\mu,\psi)$. The blade element equations are iterated until convergence for axial and tangential inductions, a_n and a'. The initial condition is made for axial and tangential inductions of $a_n(\mu) = 1/3$ and $a'(\mu) = 0$ respectively. The axial and tangential velocity, assuming negligible tilt, are (10, 11):

$$v_x(\mu,\psi) = U(\mu,\psi)(1-a_n)\cos(\gamma), \qquad [16]$$
$$v_t(\mu,\psi) = (1+a')\lambda\mu - U(\mu,\psi)(1-a_n)\cos(\psi)\sin(\gamma). \qquad [17]$$

The inflow wind speed and angle, w and ϕ are

$$w(\mu,\psi)^2 = v_x(\mu,\psi)^2 + v_t(\mu,\psi)^2, \qquad [18]$$
$$\phi(\mu,\psi) = \tan^{-1}\left(\frac{v_x(\mu,\psi)}{v_t(\mu,\psi)}\right). \qquad [19]$$

The angle of attack is

$$\alpha(\mu,\psi) = \phi(\mu,\psi) - \theta_t(\mu) - \theta_p, \qquad [20]$$

and the rotor normal and tangential force coefficients are

$$C_n(\mu,\psi) = \cos(\phi)C_l(\mu,\alpha(\mu,\psi)) + \sin(\phi)C_d(\mu,\alpha(\mu,\psi)), \qquad [21]$$
$$C_{tan}(\mu,\psi) = \sin(\phi)C_l(\mu,\alpha(\mu,\psi)) - \cos(\phi)C_d(\mu,\alpha(\mu,\psi)). \qquad [22]$$

The thrust coefficient, $C_T(\mu,\psi)$ is

$$C_T(\mu,\psi) = \sigma(\mu)w(\mu,\psi)^2 C_n(\mu,\psi). \qquad [23]$$

Tip and root losses are included in the blade element model in the standard approach (12) where the thrust coefficient is modified as

$$C_T^{corr}(\mu,\psi) = \frac{C_T(\mu,\psi)}{F_{\text{tip}}(\mu)F_{\text{root}}(\mu)}. \qquad [24]$$

The tip loss function is (13)

$$F_{\text{tip}}(\mu) = \frac{2}{\pi}\arccos\left(\exp\left(-\frac{N(1-\mu)}{2\mu\sin\phi}\right)\right), \qquad [25]$$

and the root loss function is

$$F_{\text{root}}(\mu) = \frac{2}{\pi}\arccos\left(\exp\left(-\frac{B(\mu - R_{\text{hub}}/R)}{2\mu\sin\phi}\right)\right), \qquad [26]$$

in which R_{hub} is the radius of the rotor hub. The rotor-normal and tangential induction factors are initialized with $a_n(\mu,\psi) = 1/3$ and $a'(\mu,\psi) = 0$, respectively. At each iteration, Eqs 16-24 are solved to update the rotor normal and tangential forces based on the current values of $a_n(\mu,\psi)$ and $a'(\mu,\psi)$. The blade element model is connected to the Unified Momentum Model accounting for blockage by solving Eq. 9-14 with C_T^{corr}, γ, and $\beta = 0.094$ as inputs to update $a_n(\mu,\psi)$. The tangential induction factor is updated using (4)

$$a'(\mu,\psi) = \frac{\sigma(\mu)C_{tan}(\mu,\psi)w^2(\mu,\psi)}{4\lambda\mu F(\mu)(1-a_n)\cos(\gamma)u_\infty^2}. \qquad [27]$$

After the calculation of the new rotor-normal and tangential induction factors, $a_n(\mu,\psi)$ and $a'(\mu,\psi)$, respectively, we return back to Eq. 16 to restart the loop. The coupled system is solved using fixed point iteration with relaxation until converged

solutions for $a_n(\mu,\phi)$ and $a'(\mu,\psi)$ are reached. The equations are solved on a polar grid with 29 radial points and 36 azimuthal points. The rotor-averaged power coefficient is calculated by integrating the local power coefficient over the rotor area (14),

$$\bar{C}_P = \frac{1}{\pi}\int_0^1\int_0^{2\pi} \lambda\mu^2\sigma(\mu)w(\mu,\psi)^2 C_{tan}(\mu,\psi)d\psi d\mu. \quad [28]$$

Similarly, the rotor averaged thrust coefficient is

$$\bar{C}_T = \frac{1}{\pi}\int_0^1\int_0^{2\pi} \sigma(\mu)w(\mu,\psi)^2 C_n(\mu,\psi)\mu d\psi d\mu. \quad [29]$$

The streamwise and lateral outlet velocities are be calculated using the rotor-averaged thrust coefficient $\bar{C}_T$ in the Unified Momentum Model $[\bar{a}_n, \bar{u}_4, \bar{v}_4] = f_{\text{Unified}}(\bar{C}_T, \gamma)$.

Validation and sensitivity analysis. We validate the open-source MITRotor code used in this study to solve the Unified Wind Turbine Model (UWT) blade element momentum (BEM) model against CCBlade (15), an established and widely-used steady-state BEM solver, by reproducing the thrust and power curves for four IEA reference turbine models in Figure S4. To ensure consistency of the models used in MITRotor and CCBlade in the validation, we use the empirical Buhl high thrust correction within both codes. These results demonstrate that MITRotor yields predictions that are consistent with state-of-practice BEM solvers.

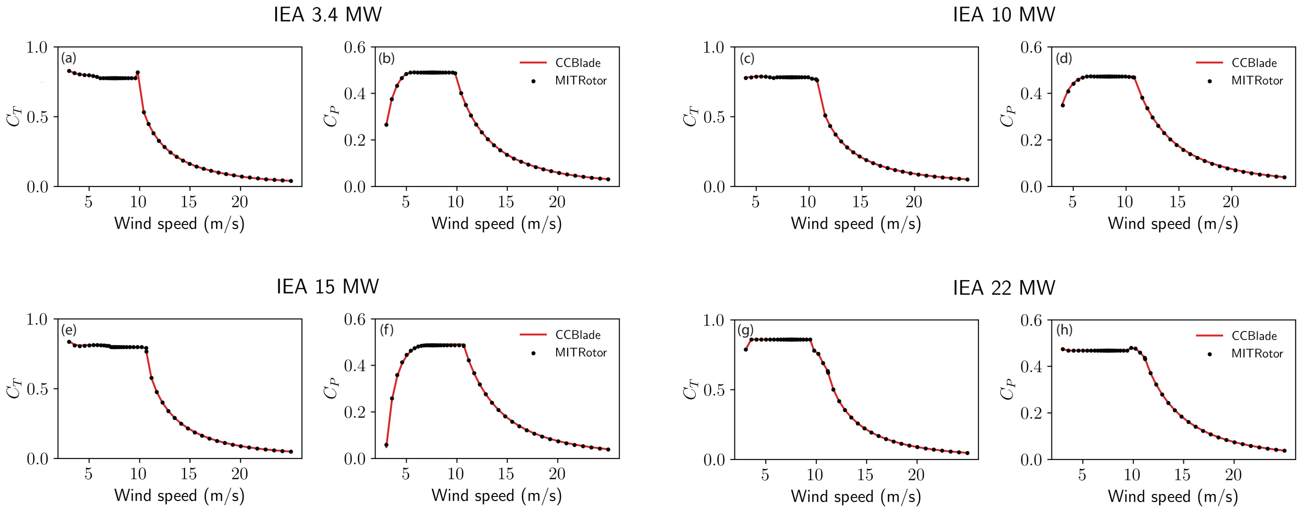

Fig. S4. Comparison of MITRotor with reference wind turbine power curves generated using CCBlade. (a) Power coefficient and (b) thrust coefficient of the IEA 3.4 MW reference wind turbine generated using the steady-state BEM solver CCBlade and MITRotor. (c,d) Same for IEA 10 MW reference wind turbine. (e,f) Same for IEA 15 MW reference wind turbine. (g,h) Same for IEA 22 MW reference wind turbine.

Beyond the momentum modeling improvements provided by the Unified Momentum Model described in the main text, there are several sources of uncertainty in the blade element modeling portion of BEM. A key uncertainty in blade element models is the reduction in blade forces near the tip arising from tip vortices (13), which is usually addressed with empirical tip-loss modeling. The effect of these tip losses has been shown to vary widely depending on the blade geometry and operating conditions (16). To illustrate the uncertainty in power and thrust coefficients, we solve the model including no tip losses, a standard Prandtl tip loss correction, and the empirical tip loss correction term of (16). For the specific turbine tested in the HRTF, there has not been a detailed investigation on the tip-losses and their associated modeling. In Figure S5, we show the yaw-aligned power (C_P) and thrust coefficient (C_T) predictions of the Unified Wind Turbine (UWT) model compared with the experimental results from the HRTF. The shaded region indicates the full variation in model output obtained by varying the tip loss model and toggling the tangential induction submodel on or off. We note that these tests did not include a model for the non-uniformity in the induction factor about its mean value associated with yaw misalignment (17), because this non-uniformity tends to have a relatively minor impact on the rotor-averaged thrust and power coefficients. Due to the confinement (blockage) effect in the HRTF experiments, it is possible to exceed the Betz limit value for the coefficient of power. This confinement effect is included in the UWT model used here. The high efficiencies associated with the upper bound of the uncertainty interval are from model realizations that neglect tip-losses. As shown in Figure S5, the magnitude of the C_P and C_T from BEM models is sensitive to these modeling choices.

In contrast, in Figure S6, we plot the power and thrust coefficients normalized by their yaw-aligned value including the sensitivity to the model setup. Similarly to Figure S5, we also present the uncertainty around the UWT predictions associated with the tip loss and tangential induction models, but notably, the uncertainty from these modeling choices is not visible in the

plot. These results show that when normalizing by the yaw-aligned value, the spread in predictions from different choices in the tip loss and tangential induction submodels are negligible. This reveals that the model setup has a negligible impact on the normalized power and thrust coefficients.

Therefore, to summarize the results in this section, we have demonstrated the MITRotor is consistent with state-of-practice BEM solvers, that unnormalized C_P and C_T are sensitive to empirical submodels in the blade element side of BEM not addressed nor modified in this study, but that the power and thrust coefficients that are normalized by their yaw aligned value, $C_P/C_P(\gamma = 0°)$ and $C_T/C_T(\gamma = 0°)$, are insensitive to these empirical blade element corrections. Thus, in the main text, we focus on presenting comparisons between the HRTF experiments and the UWT that are normalized, and future work should focus on reducing uncertainty and error associated with corrections used in blade element modeling. The primary investigation of this study, which is related to the power-yaw relationship modeling and the joint optimization of yaw, pitch, and tip-speed ratio, are less sensitive to numerical and modeling aspects of blade element modeling.

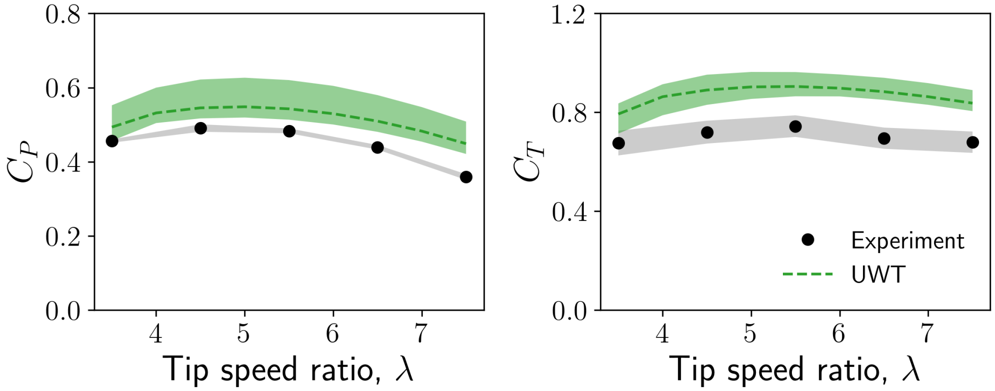

Fig. S5. Comparison of yaw-aligned ($\gamma = 0$) model predictions with experimental results for (a) power coefficient and (b) thrust coefficient across tip speed ratios. The shaded green region indicates the full variation in model output obtained by varying the tip loss correction and toggling the tangential induction submodel on or off.

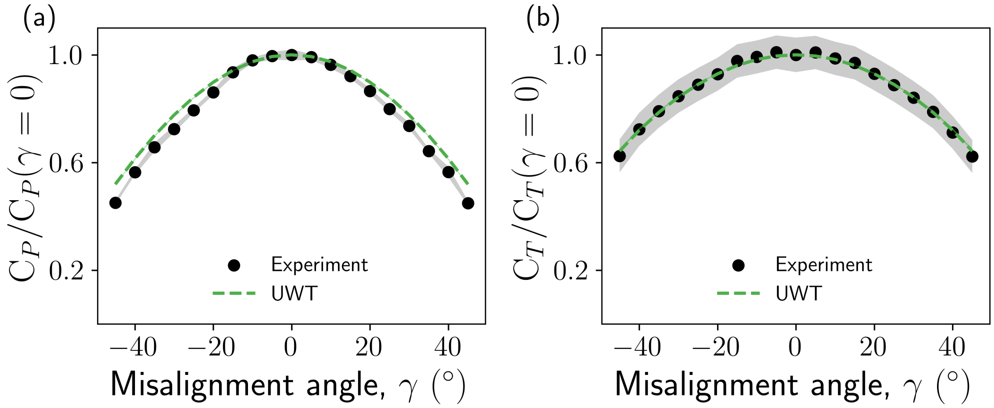

Fig. S6. Comparison of model predictions with experimental results for (a) normalized power coefficient and (b) normalized thrust coefficient across misalignment angles at $\lambda = 4.5$. The full variation in model output obtained by varying the tip loss correction and toggling the tangential induction submodel on or off is plotted as a shaded green region. The sensitivity shading is not easily visible as it is narrower than the dashed line width.

Wind farm flow control modeling and optimization.

Wind farm flow modeling. To study the impact of the Unified turbine model on the annual energy production (AEP) gain of a utility-scale wind farm, the wind farm control case study of Simley *et al.* (18) is replicated, except the empirical cosine rotor model is replaced with the Unified Wind Turbine model. The wakes in the wind farm are modeled using the exact methods of Simley *et al.* (18) and are described here for completeness.

The wake model is provided initial conditions for the wake flow from the Unified Wind Turbine model. Specifically, the wake model is provided the thrust coefficient C_T, predicted by the Unified Wind Turbine model. To accelerate the optimization, the blade element equations are solved in a rotor-averaged sense where the induction field is computed from the rotor averaged thrust as $[\bar{a}, \bar{u}_4, \bar{v}_4] = f_{\mathrm{Unified}}(\bar{C}_T, \gamma)$. The Gauss Curl Hybrid (19) wake model is used which predicts the wind speed deficit downwind of a wind turbine under arbitrary control setpoints including the influences of secondary steering and enhanced

recovery due to yaw-misalignment. The wake deficit generated by turbine i with yaw angle, γ, and thrust coefficient, C_T, is

$$\frac{\Delta u_i(x,y,z)}{u_\infty} = C_i(x)\exp\left(-\frac{(y-\delta_i(x))^2}{2\sigma_{y,i}^2(x)} - \frac{(z-z_h)^2}{2\sigma_{z,i}^2(x)}\right). \qquad [30]$$

The centerline wake deficit is

$$C(x) = 1 - \sqrt{1 - \frac{\sigma_{y0}\sigma_{z0}M_0}{\sigma_y(x)\sigma_z(x)}}, \qquad [31]$$

where $M_0 = C_0(2 - C_0)$ and $C_0 = 1 - \sqrt{1 - C_T}$ is the centerline wake deficit at the end of the near wake. The initial spanwise and vertical wake widths are

$$\frac{\sigma_{z0}}{D} = \frac{1}{2}\sqrt{\frac{u_R}{u_\infty + u_0}} \qquad [32]$$

and

$$\sigma_{y0} = \sigma_{z0}\cos(\gamma) \qquad [33]$$

where $u_R/u_\infty = \frac{1}{2}C_T\cos(\gamma)/\left(1 - \sqrt{1 - C_T\cos(\gamma)}\right)$ is the wind velocity at the rotor and $u_0/u_\infty = \sqrt{1 - C_T}$ is the velocity at the end of the near wake region. The normalized near-wake length is

$$\frac{x_0}{D} = \frac{\cos(\gamma)(1 + \sqrt{1 - C_T})}{\sqrt{2}(\alpha^* I + \beta^*(1 - \sqrt{1 - C_T}))}, \qquad [34]$$

where $\alpha^* = 2.32$ and $\beta^* = 0.154$, and I is the local turbulence intensity at the rotor. The wake deflection, $\delta(x)$, is given by

$$\frac{\delta(x)}{D} = \delta_0 + \frac{\theta_{c0}E_0}{5.2}\sqrt{\frac{\sigma_{y0}\sigma_{z0}}{k_y k_z M_0}}\log\left[\frac{(1.6 + \sqrt{M_0})\left(1.6\sqrt{\frac{\sigma_y(x)\sigma_z(x)}{\sigma_{y0}\sigma_{z0}}} - \sqrt{M_0}\right)}{(1.6 - \sqrt{M_0})\left(1.6\sqrt{\frac{\sigma_y(x)\sigma_z(x)}{\sigma_{y0}\sigma_{z0}}} + \sqrt{M_0}\right)}\right] \qquad [35]$$

where $E_0 = C_0^2 - 3\exp(\frac{1}{12})C_0 + 3\exp(\frac{1}{3})$, $\theta_{c0} = (0.3\gamma/\cos(\gamma))(1 - \sqrt{1 - C_T\cos(\gamma)})$, and $\delta_0 = x_0\tan(\theta_{c0})$. The spanwise and vertical wake widths are assumed to expand linearly as

$$\sigma_y(x) = k_y(x - x_0) + \sigma_{y0} \qquad [36]$$

$$\sigma_z(x) = k_z(x - x_0) + \sigma_{z0} \qquad [37]$$

with spanwise and vertical wake spreading rates of $k_y = k_z = 0.38I_i + 0.004$, where I_i is the local turbulence intensity at turbine i. The centerline wake-added turbulence intensity of each turbine is modeled as

$$I_+(x) = 0.5\ a_n^{0.8}\ I_0^{-0.1}\ (x/D)^{-0.32}, \qquad [38]$$

where I_0 is the ambient turbulence intensity (19). Following Niayifar *et al.* (20), the turbulence contribution from each wake is computed using the overlap area, A_w, of the downstream rotor j and twice the wake width and the maximum contribution of all upstream turbines i is taken,

$$I_{+max,j} = \max_i\left(\frac{4A_{w,ij}}{\pi D^2}I_+(x_j - x_i)\right). \qquad [39]$$

The ambient and wake-added turbulence contributions are summed as $I_j = \sqrt{I_0^2 + I_{+max,j}^2}$. The wakes are combined using the quadratic freestream superposition method proposed by Katic et al. (21),

$$u(x,y,z) = u_\infty - \sqrt{\sum_{i \in x_i < x}\Delta u_i^2(x,y,z)} \qquad [40]$$

where u is the streamwise velocity, $\Delta u_i(x,y,z)$ is the wake deficit at the location of interest generated by turbine i, and the sum is taken over all turbines upstream of the location of interest. The streamwise velocity at each rotor is estimated using a cubic average over a grid of M points over the rotor

$$\overline{u} = \left(\frac{1}{M}\sum_{i=1}^{M}u_i^3\right)^{1/3} \qquad [41]$$

where the regular square grid spans from $-D/4 < y < D/4$ and $-D/4 < z < D/4$ and $M = 25$. The rotor-averaged spanwise and vertical velocities are computed using a linear average. To capture the secondary effects of wake steering, the circulation of the counter-rotating vortices at the top and bottom of the rotor due to yaw-misalignment are modeled as

$$\Gamma_{\text{top}} = \frac{\pi}{8}C_T D\sin(\gamma)\cos(\gamma)u_\infty \qquad [42]$$

$$\Gamma_{\text{bottom}} = -\Gamma_{\text{top}} \tag{43}$$

and the circulation due to wake rotation is

$$\Gamma_{\text{wake rotation}} = \frac{\pi(a - a^2)u_\infty D}{\lambda} \tag{44}$$

where $a = (1 - \sqrt{1 - C_T \cos(\gamma)})/(2\cos(\gamma))$ is the rotor-averaged induction and λ is the tip-speed ratio. The spanwise and vertical velocities induced by a vortex of circulation Γ are

$$v = \frac{z\ \Gamma}{2\pi(y^2 + z^2)}\left[1 - \exp\left(\frac{-y^2 - z^2}{\epsilon^2}\right)\right] \tag{45}$$

and

$$w = -\frac{y\ \Gamma}{2\pi(y^2 + z^2)}\left[1 - \exp\left(\frac{-y^2 - z^2}{\epsilon^2}\right)\right] \tag{46}$$

respectively where $\epsilon = 0.2D$, and y and z are measured relative to the vortex center. The decay of the vortex-induced velocities is

$$d(x) = \frac{\epsilon^2}{4\nu_T(x - x_0)/u_\infty + \epsilon^2} \tag{47}$$

where $\nu_T = l_m^2|\frac{\partial u}{\partial z}|$ is the eddy viscosity modeled using a mixing length model. The mixing length is $l_m = \kappa z/(1 + \kappa z/\lambda_T)$ where $\kappa = 0.41$ and $\lambda_T = D/8$. Therefore, the total spanwise and vertical vortex induced velocities generated by turbine i are

$$\Delta v_i = d(x)\left(v_{\text{top}} + v_{\text{bottom}} + v_{\text{wake rotation}} + v'_{\text{top}} + v'_{\text{bottom}} + v'_{\text{wake rotation}}\right) \tag{48}$$

and

$$\Delta w_i = d(x)\left(w_{\text{top}} + w_{\text{bottom}} + w_{\text{wake rotation}} + w'_{\text{top}} + w'_{\text{bottom}} + w'_{\text{wake rotation}}\right) \tag{49}$$

where the prime ($'$) notation indicates mirrored vortices to model the influence of ground effects. The total spanwise and vertical velocities at turbine i are computed by summing the contributions from all upstream turbines as

$$v_i = \sum_j \Delta v_j \tag{50}$$

and

$$w_i = \sum_j \Delta w_j. \tag{51}$$

Secondary wake steering is modeled by computing the equivalent yaw angle, γ_{eq}, which gives the same rotor-averaged spanwise velocity as induced by the vortices of upstream turbines. The effective yaw angle provided to the wake deflection model in Eqn. 35 is then the sum of the contributions from the upstream and direct contributions, $\gamma_{eff} = \gamma_{eq} + \gamma$. To model the enhanced wake recovery due to yaw-misalignment, the added effective turbulence intensity due to yaw-misalignment is modeled as

$$I_{\text{yaw added}} = \frac{\sqrt{2k_{\text{total}}/3}}{u_i} - I \tag{52}$$

where the total turbulent kinetic energy, k_{total} is

$$k_{\text{total},i} = \frac{1}{2}\left(\overline{u'u'}_i + v_i^2 + w_i^2\right) \tag{53}$$

and $\overline{u'u'}_i = 3u_i^2 I_i^2$ are the time-averaged turbulent fluctuations at turbine i. The total effective turbulence intensity is then $I_{\text{eff}} = I + 2I_{\text{yaw added}}$

Wind farm control optimization. We compare the joint control method enabled by the Unified Wind Turbine model with a baseline individual control case, and a state-of-practice cosine control method. In the baseline case, turbines operate greedily to maximize their individual power output. In the cosine control case, the turbine yaw angles are optimized using an empirical cosine model for the thrust-yaw and power-yaw relationships and then prescribed to a turbine with fixed pitch angle and tip speed ratio set by a $k - \Omega^2$ torque controller. Finally, in the joint control case the pitch angles, tip speed ratios, and yaw angles are co-optimized using the novel predictive capability of the Unified Wind Turbine model.

In the baseline case, all turbines are yaw-aligned and operate at the individually optimal pitch angle and tip speed ratio. The power of a single yaw-aligned turbine is maximized to determine the individually optimal pitch angle, $\theta_p^* = 0.2124°$ and tip-speed ratio, $\lambda^* = 8.129$. In the cosine control case, empirical cosine models for the power-yaw and thrust-yaw relationships are used to maximize wind farm power by optimizing the turbine yaw angles. The state-of-practice cosine rotor model empirically estimates the power-yaw and thrust-yaw relationships as

$$\frac{P(\gamma)}{P(\gamma = 0)} = \cos^{P_p}(\gamma) \tag{54}$$

and

$$\frac{T(\gamma)}{T(\gamma=0)} = \cos^{T_p}(\gamma) \quad [55]$$

where P and T are the rotor thrust and power respectively. The exponents, P_p and T_p, are typically fit to field measurements of yaw-misaligned wind turbines. The cosine model does not allow for co-optimization of the yaw-misalignment angle with the pitch angle and tip-speed ratio and can also introduce model value error through the choices of T_p and P_p. To reveal the influence of both of these factors, three cosine model control optimizations are performed, one using the truth values ($T_p = 1.03$ and $P_p = 1.49$) and two with standard choices of the model values ($T_p = 2$ and $P_p = 2$, and $T_p = 2$ and $P_p = 3$). To replicate the behavior of a yaw-misaligned wind turbine in the absence of a more advanced strategy, the tip speed ratio changes with misalignment according to a standard $k\ \Omega^2$ torque controller. Specifically, the turbine torque is controlled so that $Q = k\ \Omega^2$ where Q is the rotor torque, k is the proportionality constant chosen to maximize the yaw-aligned turbine power and Ω is the rotational rate of the turbine (22). This controller determines the rate at which the tip speed ratio decreases in yaw-misalignment. To allow for direct comparison, the optimized yaw values are then prescribed to the BEM rotor model in $k-\Omega^2$ control by assigning the θ_p^* and $\lambda(\gamma) = \lambda(\gamma=0)\cos^{\Lambda_p}(\gamma)$ where $\Lambda_p = 0.498$ is fit to a single misaligned turbine in $k-\Omega^2$ control. Finally, the joint control optimization is performed by simultaneously optimizing the pitch angle, tip-speed ratio, and yaw-misalignment angle, of each turbine in the wind farm for each wind direction using the Unified Wind Turbine model.

The IEA 3.4 MW reference turbine model (23) is used for the wind farm control optimizations. To match the rotor diameter, $D = 90$ m, and rated power of the Vestas 1.8 MW turbine at the Glacier Hills Wind Farm, the rated wind speed is computed following (18) as

$$u_{\text{rated}} = \left(\frac{2P_{\text{rated}}}{\rho A_d C_p^*}\right)^{1/3} = 10.21\ \text{m s}^{-1} \quad [56]$$

where $P_{\text{rated}} = 1.8$MW is the rated power of the case study turbine model, $\rho = 1.225$ kg/m^3 is the density of dry air and $C_p^* = 0.434$ is the optimal power coefficient of the BEM rotor model. Above the rated wind speed, no wake steering is applied. Instead, all turbines are yaw-aligned ($\gamma = 0$) and controlled using a standard Region III control strategy in which the rotational rate of the turbine is fixed at the rated value and the pitch angle is increased such that the power remains at the rated value (23). All of the optimizations are performed using the SciPy sequential least squares quadratic programming (SLSQP) function and the gradients are computed using the Dualitic automatic-differentiation package.

Wind farm control optimization case study setup. The wind farm control case study is performed at the Glacier Hills Wind Farm in Wisconsin, USA. The wind farm layout is publicly available at the U.S. Wind Turbine Database (24). Three years (2018-2020) of debiased ERA5 wind speed and direction timeseries data for the Glacier Hills Wind Farm are used as in (18, 25). A constant ambient turbulence intensity of $I = 9\%$ is used for all evaluations of the wind farm model. Following (18), the yaw angles are constrained within the range $-25° \leq \gamma \leq 25°$. The wind farm model is used to evaluate the wind farm power using a wind speed resolution of 0.5 m/s and a wind direction resolution of 1°. The wind farm power is then computed for the entire three year timeseries by linear interpolation of the model results.

Comparison of model predictions with Tamaro *et al.* (2024)

We now compare the predictions of the UWT model against the model of Tamaro *et al.* (26). Predictive models for wind turbines consist of an induction model which captures the induced velocities of the rotor and a method for predicting the thrust force of the rotor given these induced velocities. The Tamaro *et al.* (2024) model differs from the UWT model presented in this work both in its induction closure and thrust model. For the induction closure, the Tamaro *et al.* (26) induction model accounts for misalignment, yielding the same induction model as Heck *et al.* (27), but does not account for high thrust operation captured in the Unified Momentum Model (4) nor confined operation (6). To determine the thrust force given the induced velocity, the Tamaro *et al.* (2024) model uses a simplified blade element model in order to be computationally efficient. In this way, the Tamaro *et al.* (2024) model provides predictions of thrust and power as a function of tip speed ratio and pitch angle directly without the need for an iterative blade element momentum solution. As a result, the Tamaro *et al.* (2024) model is faster to evaluate than the UWT model but incorporates less induction and blade aerodynamics physics.

Description and setup of the Tamaro *et al.* (2024) model. The Tamaro *et al.* (26) model is formulated using the blade element momentum (BEM) equations and making targeted simplifications including linearizing the airfoil lift force and assuming a single representative value of the drag coefficient C_D, lift slope $C_{L,\alpha}$, and blade twist β for the entire blade. In Tamaro *et al.* (26), the representative values of C_D, $C_{L,\alpha}$, and β are chosen by fitting them to the validation dataset. As an alternative to calibration on the validation dataset, Tamaro *et al.* (26) recommends taking the values of C_D, $C_{L,\alpha}$, and β at 2/3 span (r/R = 2/3). To perform a side-by-side predictive test of the Tamaro *et al.* (26) model compared to the fully predictive UWT which has no such tunable parameters, we use this method for choosing the representative parameter values. In this way, both models are required to predict the experimental observations without receiving any *a priori* knowledge of the validation dataset. The values of the parameters used are given here:

- Drag coefficient at 2/3 span and zero angle of attack: $C_D = 0.0092$
- Lift slope at 2/3 span and zero angle of attack, including 2/3 scaling factor: $2C_{L,\alpha}/3 = 4.5560$

- Twist at 2/3 span: $\beta = 2.65°$
- Blade solidity at 2/3 span: $\sigma = 0.1294$
- Shear coefficient: $k = 0$
- Tilt angle: $\delta = 0°$
- Blade pitch angle: $\theta_p = 5°$

Comparison of model predictions. First, we reproduce Figure 2 from the main text of the paper, where we compare the models to the HRTF thrust and power coefficient results at a tip-speed ratio of 4.5. As can be seen in Figure S7, the model of Tamaro *et al.* (26) captures the correct qualitative trend, but exhibits higher error than the UWT for both normalized thrust and power coefficients for all misalignment angles.

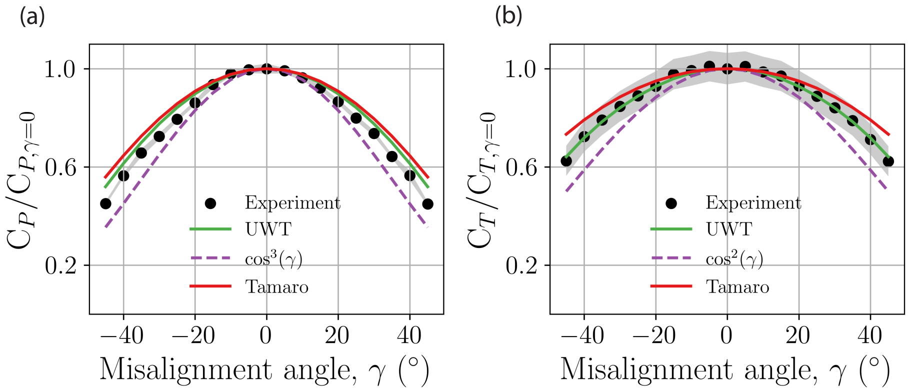

Fig. S7. Comparison of the Tamaro *et al.* (2024) (26) model and the present UWT model predictions with experimental measurements for normalized (a) thrust and (b) power at a near-optimal tip-speed ratio of $\lambda = 4.5$.

In Figure S8, we reproduce Figure 3 from the main text and add the model predictions of Tamaro *et al.* (26) for comparison. The results demonstrate that the Tamaro *et al.* (2024) model is able to predict the normalized optimal power coefficient accurately, similar to the UWT, but exhibits larger structural differences in the optimal tip-speed ratios that achieve this optimal power, as shown in Figure S8(a, b). While both models exhibit some quantitative error in the prediction of the most accurate cosine exponent for the power (Figure S8(c)), the Tamaro *et al.* (26) model does not reproduce the dependency of the cosine exponent P_p on the tip-speed ratio. Similar results are reported for the thrust coefficient cosine exponent, except for the thrust coefficient, the UWT exhibits small quantitative error.

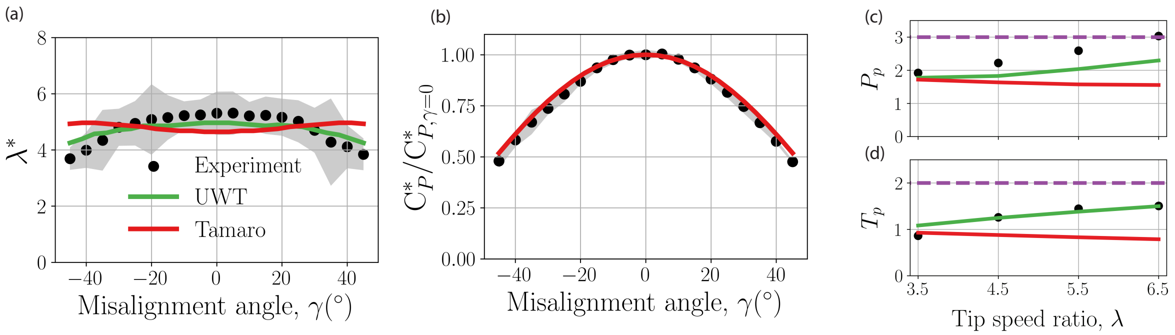

Fig. S8. The (a) power-maximizing tip speed ratio as a function of misalignment angle and (b) optimized power as a function of misalignment angle are shown for each model and compared with the experimentally determined values. Additionally, the (c) power and (d) thrust losses with misalignment across tip speed ratios are characterized by fitting T_p and P_p respectively using a cosine model.

SI Dataset S1 (expDataNominal.csv)

Experimental results of λ, C_T and C_P including absolute uncertainty.

SI Dataset S2 (expDataOptimal.csv)

Experimental results of λ^*, C_T^* and C_P^* including absolute uncertainty.

References

1. MA Miller, J Kiefer, C Westergaard, MOL Hansen, M Hultmark, Horizontal axis wind turbine testing at high Reynolds numbers. *Phys. Rev. Fluids* **4**, 110504 (2019).
2. RJ Moffat, Contributions to the Theory of Single-Sample Uncertainty Analysis. *J. Fluids Eng.* **104**, 250–258 (1982).
3. RJ Moffat, Describing the uncertainties in experimental results. *Exp. Therm. Fluid Sci.* **1**, 3–17 (1988).
4. J Liew, KS Heck, MF Howland, Unified momentum model for rotor aerodynamics across operating regimes. *Nat. Commun.* **15**, 6658 (2024).
5. GA Van Kuik, The lanchester–betz–joukowsky limit. *Wind. Energy: An Int. J. for Prog. Appl. Wind. Power Convers. Technol.* **10**, 289–291 (2007).
6. IML Upfal, et al., An analytical model for rotors in confined flow across operating regimes (2026) arXiv preprint https://arxiv.org/abs/2603.06895.
7. H Ross, B Polagye, An experimental assessment of analytical blockage corrections for turbines. *Renew. Energy* **152**, 1328–1341 (2020).
8. C Garrett, P Cummins, The efficiency of a turbine in a tidal channel. *J. fluid mechanics* **588**, 243–251 (2007).
9. O Ndindayino, A Puel, J Meyers, Effect of blockage on wind turbine power and wake development. *Wind. Energy Sci. Discuss.* **2025**, 1–24 (2025).
10. MF Howland, et al., Influence of atmospheric conditions on the power production of utility-scale wind turbines in yaw misalignment. *J. Renew. Sustain. Energy* **12**, 063307 (2020).
11. KA Kragh, MH Hansen, Load alleviation of wind turbines by yaw misalignment. *Wind. Energy* **17**, 971–982 (2014).
12. HA Madsen, TJ Larsen, GR Pirrung, A Li, F Zahle, Implementation of the blade element momentum model on a polar grid and its aeroelastic load impact. *Wind. energy science* **5**, 1–27 (2020).
13. E Branlard, Wind turbine aerodynamics and vorticity-based methods: Fundamentals and recent applications (2017).
14. T Burton, N Jenkins, D Sharpe, E Bossanyi, *Wind Energy Handbook.* (John Wiley & Sons), (2011).
15. S Ning, Ccblade documentation: Release 0.1. 0, (National Renewable Energy Lab.(NREL), Golden, CO (United States)), Technical report (2013).
16. D Maniaci, S Schmitz, Extended Glauert Tip Correction to Include Vortex Rollup Effects. *J. Physics: Conf. Ser.* **753**, 022051 (2016).
17. C Hur, T Berdowski, C Simao Ferreira, K Boorsma, G Schepers, A review of momentum models for the actuator disk in yaw in *AIAA Scitech 2019 Forum.* p. 1799 (2019).
18. E Simley, D Millstein, S Jeong, P Fleming, The value of wake steering wind farm flow control in us energy markets. *Wind. Energy Sci.* **9**, 219–234 (2024).
19. J King, et al., Control-oriented model for secondary effects of wake steering. *Wind. Energy Sci.* **6**, 701–714 (2021).
20. A Niayifar, F Porté-Agel, Analytical Modeling of Wind Farms: A New Approach for Power Prediction. *Energies* **9**, 741 (2016).
21. I Katic, J Højstrup, N Jensen, A simple model for cluster efficiency. *EWEC'86. Proceedings. Vol. 1* (1987).
22. EA Bossanyi, The design of closed loop controllers for wind turbines. *Wind. Energy* **3**, 149–163 (2000).
23. P Bortolotti, et al., Iea wind task 37 on systems engineering in wind energy – wp2.1 reference wind turbines, (International Energy Agency), Technical report (2019).
24. B Hoen, et al., United states wind turbine database, (U.S. Geological Survey, American Clean Power Association, and Lawrence Berkeley National Laboratory), Technical report (2025).
25. E Simley, D Millstein, S Jeong, P Fleming, Data from "the value of wake steering wind farm flow control in us energy markets." *Zenodo.* available at https://doi.org/10.5281/zenodo.10493111. deposited january 2024. (2024) Data from "The value of wake steering wind farm flow control in US energy markets." *Zenodo.* Available at https://doi.org/10.5281/zenodo.10493111. Deposited January 2024.
26. S Tamaro, F Campagnolo, CL Bottasso, On the power and control of a misaligned rotor–beyond the cosine law. *Wind. Energy Sci.* **9**, 1547–1575 (2024).
27. KS Heck, HM Johlas, MF Howland, Modelling the induction, thrust and power of a yaw-misaligned actuator disk. *J. Fluid Mech.* **959**, A9 (2023).
